# Nucleic Acid‐Locked Smart Carrier for Photothermal/Chemotherapy‐Amplified Immunogenic Cell Death to Enhance Systemic Antitumor Efficacy

**DOI:** 10.1002/advs.202503299

**Published:** 2025-04-04

**Authors:** Jiayang Zhang, Tianyu Gai, Jiwei Wang, Yucai Wu, Si‐Ming Zeng, Dongyuan Zhao, Wei Li

**Affiliations:** ^1^ Department of Chemistry Laboratory of Advanced Materials Shanghai Key Laboratory of Molecular Catalysis and Innovative Materials Fudan University Shanghai 200433 P. R. China; ^2^ Key Laboratory of Biomedical Polymers of Ministry of Education & Department of Chemistry Wuhan University Wuhan 430072 P. R. China

**Keywords:** combination therapy, gold nanorod, immune activation, mesoporous silica, nucleic acid

## Abstract

Immunotherapy holds great promise in the fight against cancer; however, it often encounters poor immunogenicity with limited therapeutic efficacy. Combining multiple treatment modalities provides a trustworthy strategy for achieving a robust antitumor effect. In this study, a nucleic acid‐locked smart carrier (NASC) is developed to amplify immunogenic cell death (ICD) through the synergistic integration of photothermal therapy (PTT) and chemotherapy for high‐performance monotherapy. Mesoporous silica‐coated gold nanorod (MSGNR) serves as the reservoir for anticancer drug doxorubicin (DOX) and is capped with a sequence‐specific duplex unit containing a tumor‐specific targeting AS1411 fragment, resulting in the formation of NASC. With AS1411 targeting, the NASC can specifically target and be internalized into tumor cells with high expression of nucleolin, where the duplex capping can be unlocked by the intracellularly overexpressed adenosine triphosphate. Subsequently, the released DOX synergized with MSGNR‐mediated PTT following laser irradiation induces direct cell killing, which, concurrently, triggers ICD to activate antineoplastic immunity with an increased number of T lymphocytes. This triple‐collaborative strategy, further combined with anti‐programmed death‐1 antibody (αPD‐1)‐mediated immune checkpoint blockade (ICB) therapy, shows a robust therapeutic efficacy in both unilateral and bilateral tumor models.

## Graphical abstract

Multiple therapeutic modalities combined in a synergistic way that can modulate different immune axes are demonstrated potentially effective in boosting immunotherapy. A nucleic acid‐locked smart carrier (NASC) performed as an “all‐in‐one” nanoplatform is constructed for efficient eliciting chemo‐ and photothermal‐amplified immunogenic cell death to synergistically achieve robust antitumor efficacy.

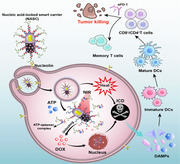

## 1 Introduction

Immunotherapy has arisen as a powerful therapeutic modality for cancer treatment.^[^ ^1^ ^]^ Despite significant progress in the development and assent of various immunotherapy drugs in both preclinical and clinical settings, low immunogenicity remains a major challenge, limiting the therapeutic efficacy and resulting in only a subset of patients achieving positive antitumor responses.^[^ ^2^ ^]^ Therefore, developing efficient strategies to enhance response rates is essential to improving the overall potency of immunotherapy.

Immunogenic cell death (ICD) is a key mechanism for activating antitumor immunity.^[^ ^3^ ^]^ During ICD, dying malignant cells release or expose numerous damage‐associated molecular patterns (DAMPs), e.g. calreticulin (CRT), high‐mobility group box 1 (HMGB1), and adenosine triphosphate (ATP).^[^ ^4^ ^]^ These DAMPs affect initiating both innate and adaptive immune responses. Evidence shows that therapies like radiotherapy, photothermal therapy (PTT),^[^ ^5^ ^]^ and certain chemotherapeutic agents (e.g., DNA‐damaging agents, anthracyclines) can induce ICD,^[^ ^6^ ^]^ thereby enhancing the therapeutic effects of conventional cancer treatments. However, so far, there are only a few bona fide ICD inducers as therapeutics been used successfully in the clinic. This may be ascribed to that different ICD inducers cannot elicit the DAMPs signals at the same level to activate a thorough‐paced immune response with high degrees of dendritic cells (DCs) maturation and T cell activation, especially when these inducers are used alone.^[^ ^7^ ^]^ In addition to ICD, other immunotherapies, such as immune checkpoint blockade (ICB), which directly activates antitumor responses, hold significant promise.^[^ ^8^ ^]^ However, their effectiveness is often hindered by the ability of tumor cells to escape T‐cell recognition.^[^ ^9^ ^]^ Therefore, multimodal therapy by integrating different kinds of treatment modalities presents considerable appeal in reinforcing antitumor effects, which allows different therapeutic mechanisms to work synergistically to elicit a robust immune response from multiple axes.^[^ ^10^ ^]^

In recent years, the development of “all‐in‐one” nanoplatforms has become a major focus of research. These nanoplatforms are designed to integrate multiple therapeutic agents and functionalities into a single system.^[^ ^11^ ^]^ Traditional small‐molecule therapies often suffer from severe off‐target effects, low bioavailability, poor water solubility, and rapid clearance from the body.^[^ ^12^ ^]^ Nanoparticles (NPs), however, can overcome these limitations by encapsulating drugs, enhancing tumor‐targeting capabilities, extending blood circulation time, and co‐delivering multiple therapeutics.^[^ ^13^ ^]^ A wide range of NPs have gained attention due to their favorable physicochemical properties.^[^ ^14^ ^]^ For example, gold nanorods, which generate near‐infrared (NIR) photothermal effects, and mesoporous silica NPs, known for their high drug‐loading capacity, can facilitate the “all‐in‐one” strategy.^[^ ^15^ ^]^ Despite these advantages, challenges remain in the use of NPs for combinatorial treatments, particularly in terms of biocompatibility and the need for intelligent systems capable of functioning effectively within complex biological environments.^[^ ^16^ ^]^

**Scheme 1:**
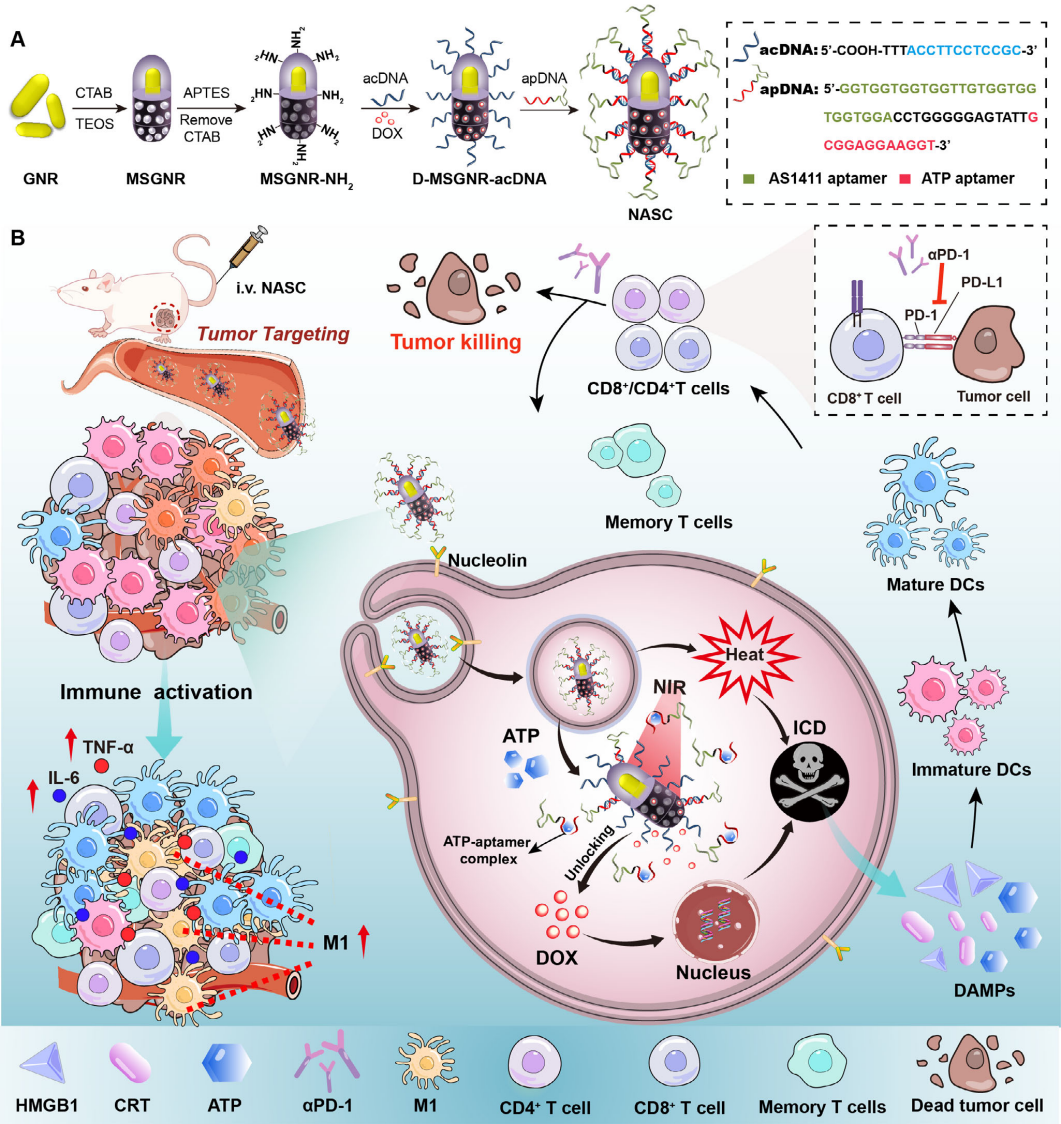
Schematic illustration of the nucleic acid‐locked smart carrier (NASC) for efficiently eliciting chemo‐ and photothermal‐amplified immunogenic cell death to synergistically achieve effective antitumor efficacy.

In this study, a biologically compatible “all‐in‐one” nano platform of the nucleic acid‐locked smart carrier (NASC) was designed to boost cancer immunotherapy through a combination of chemotherapy, photothermal therapy (PTT), and immunotherapy (**Scheme** **1**). The inner core of the NASC was a mesoporous silica‐coated gold nanorod (MSGNR), which incorporates a gold nanorod (GNR) core as a photothermal agent and a mesoporous shell for drug storage, loaded with chemotherapeutic drug doxorubicin (DOX), and the outer surface was modified with a sequence‐specific duplex nucleic acids (NAs) to endow the nanomaterial with biological functions (e.g., AS1411‐mediated tumor targeting and stimuli‐responsiveness).^[^ ^17^ ^]^ Owing to the excellent biocompatibility and precise editing capability, the duplex NAs formed by two complementary DNA strands were used to act as smart controllers for on‐demand drug release, which could be unlocked by specific biological substrates (i.e., ATP) in tumor cells.^[^ ^18^ ^]^ Upon specific tumor uptake and efficient intracellular drug release,^[^ ^19^ ^]^ direct cell killing could be induced by the released DOX and GNR‐mediated PTT, which, concurrently, stimulate high levels of ICD for boosting tumor immunogenicity. In combination with ICB therapy, the antitumor efficacy was dramatically potentiated against both primary and distant tumors with a robust anticancer immune response, demonstrating the considerable potential of this versatile NASC with a smart design for tumor‐targeted therapy.

## 2 Results and Discussion

### 2.1 Synthesis and Characterization of NASC

**Figure 1:**
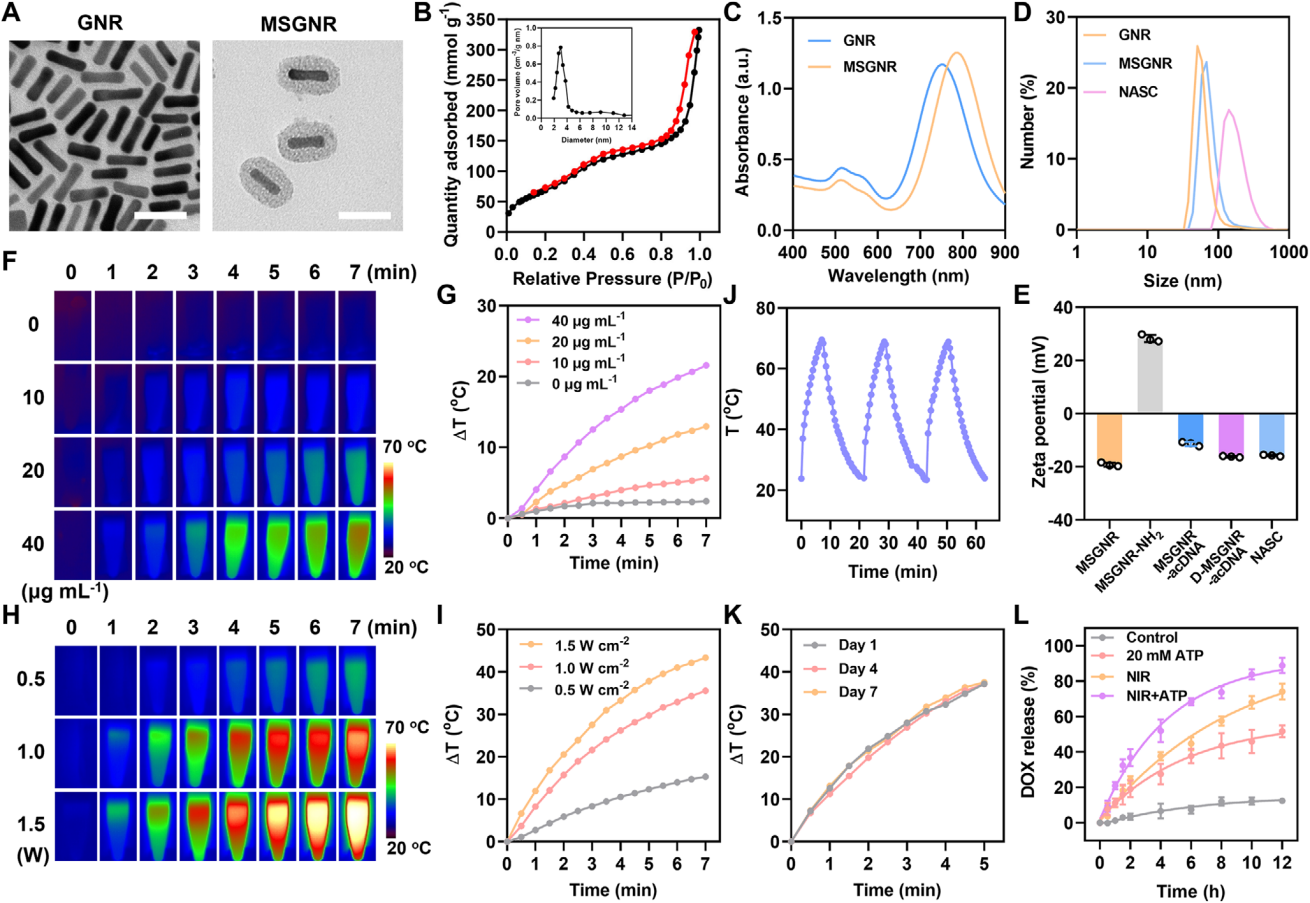
Synthesis and characterization of NASC. A) TEM images of GNR and MSGNR. Scale bar: 50 nm. B) N_2_ adsorption/desorption analysis of MSGNR. Inset: pore size distribution. C) UV–vis spectra of GNR and MSGNR. D) DLS and E) zeta potentials of different nanoparticles. F) Thermal images of various concentrations of NASC irradiated by an 808 nm laser (0.5 W cm^−2^) for 7 min, and G) the corresponding temperature change curves. H) Thermal images of NASC (20 µg mL^−1^) irradiated by various powers of 808 nm laser for 7 min, and I) the corresponding temperature change curves. J) ON/OFF switchable temperature change of NASC under cyclic laser irradiation (7 min of irradiation for each cycle). K) thermal stability of NASC in serum at days 1, 4, and 7. L) Time‐dependent DOX release from NASC under different conditions. Data were performed as the mean ± SD (*n* = 3).

Owing to the fused property of near‐infrared (NIR) laser‐triggered heat generation as well as high drug loading capacity, MSGNR was chosen as the favorable scaffold to construct nanotherapeutics. First, GNR NPs were prepared through a seed‐mediated surfactant‐directed approach,^[^ ^20^ ^]^ followed by coating the surface with a mesoporous silica shell by using a modified sol‐gel method.^[^ ^21^ ^]^ Transmission electron microscopy (TEM) images illustrated that GNR exhibited a rod‐shaped morphology with an average length and width of ≈38.7 ± 4.1 and 9.8 ± 1.5 nm (**Figure** **1**A), resulting in an aspect ratio of ≈3.9 that can ensure an efficient photothermal conversion. After silica coating, the as‐prepared MSGNR was observed with an intact core‐shell structure, in which the high‐contrast GNR was covered with a ≈12 nm mesoporous silica shell. Moreover, the Brunauer–Emmett–Teller (BET) surface area of MSGNR was determined to be 439 m^2^ g^−1^, and the pore size was ≈3 nm, confirming the existence of mesoporous structure for use as an excellent drug carrier (Figure 1B). The UV–vis light absorption spectrum showed that, as compared with bare GNR, the plasmon resonance peak of MSGNR at the transverse band remained unchanged but red‐shifted ≈53 nm at the longitudinal band to 787 nm (Figure 1C), which was still located in the NIR region to guarantee the light‐to‐heat conversion effect. To achieve multi‐functionalization, MSGNR was reacted with 3‐aminopropyltriethoxysilane (APTES), followed by treatment with a hydrochloric acid/methanol mixture to remove the pore‐embedded cetyltrimethylammonium bromide (CTAB) surfactant for obtaining MSGNR‐NH_2_. Via the condensation reaction between ─COOH and ─NH_2_, an anchoring DNA (acDNA) was modified on the surface of MSGNR‐NH_2_, and the resulting MSGNR‐acDNA was loaded with the chemotherapeutic drug DOX. The DOX‐loaded nanoparticles were locked by the hybridization of the acDNA (on the nanoparticle surfaces) with an aptamer DNA (apDNA) composed of an ATP aptamer fragment (that is complementary to the sequence of acDNA) and an AS1411 aptamer fragment, leading to the formation of duplex NA‐locked, DOX‐loaded, nanocarrier (NASC).^[^ ^22^ ^]^ Dynamic light scattering (DLS) showed that the obtained NASC still possessed uniform size and good dispersibility (polydispersity index < 0.2), just resulting in a slight increase in hydrodynamic size (Δsize: ≈50 nm) compared to the bare MSGNR (Figure 1D). Moreover, the introduction of amino groups and NAs caused a significant surface charge reversion (ζ‐potential: −19.5 mV for MSGNR, +28.3 mV for MSGNR‐NH_2_, −11.2 mV for MSGNR‐acDNA, and −16.2 mV for NASC) (Figure 1E), revealing the successful synthesis of NASC. Also, the strong negative charge of the resultant NASC would be favorable for protein resistance and contribute to good colloidal stability, which is of great avail to achieve long circulation during in vivo application. According to an established fluorescence calibration curve of DOX (Figure S1, Supporting Information), the loading amount of DOX in the nanocarrier was calculated to be 8.64% (w/w).

To confirm the photothermal conversion ability of NASC, the real‐time temperature variations of NASC solution under 808 nm laser irradiation were recorded using an IR camera. As shown in Figure 1F,G, at a specific laser power (0.5 W cm^−^^2^), the temperature increase of NASC solution was positively correlated with the NPs concentration. The higher the concentration, the more rapidly the temperature rose. It could be observed that when there were no NPs in the solution (0 µg mL^−1^), the solution did not show significant temperature changes even with a 7 min laser irradiation. Along with the increase of the NPs concentration (from 10 to 40 µg mL^−1^), the temperature rising rate grew gradually, and the most significant temperature change (ΔT ≈ 21.6 °C in 7 min irradiation) was detected at a concentration of 40 µg mL^−1^. At a fixed concentration of 20 µg mL^−1^, the temperature increased with increasing the laser power (Figure 1H,I), further demonstrating the light‐to‐heat conversion effect of NASC, which could be finely controlled by adjusting the nanoparticle concentration and NIR laser power. Such laser‐induced heating exhibits switchable ON/OFF controlled behavior. Upon laser irradiation, a rapid temperature increase was detected, which returned to a low‐temperature level when the NIR laser was withdrawn (Figure 1J). Furthermore, no significant temperature attenuation was observed during a three‐cycle laser irradiation experiment involving repeated heating and cooling phases (7 min on, 18 min off, 1.0 W cm^−2^), confirming that NASC possesses stable and robust photothermal properties. Moreover, the photothermal properties of NASC in serum remained stable over 7 days (Figure 1K).

It should be noted that in the presence of ATP, a competitive interaction would occur with the formation of ATP‐aptamer complexes, leading to the dissociation of the duplex NAs locks and the detachment of apDNA from the nanosystem.^[^ ^23^ ^]^ Moreover, the double‐strand DNA was unstable at relatively high temperatures, which can also induce the unlocking of duplex NAs for triggering drug release. Therefore, the in vitro DOX release profile from NASC was investigated at various conditions. As shown in Figure 1L, compared with the low drug release in phosphate‐buffered saline (PBS), a notably accelerated DOX release was detected in the presence of 20 mm ATP or NIR irradiation, resulting in 51.7% and 74.1% cumulative release after 12 h incubation, respectively. Under the simultaneous action of these two triggers, the release rate was further enhanced, and more than 88.8% of DOX liberation was detected. Since the concentration of ATP in tumor cells is significantly higher than that in normal cells,^[^ ^24^ ^]^ this ATP‐driven release behavior can render the tailor‐designed NASC as a smart nanosystem for on‐demand drug delivery into targeted tumor cells.

### 2.2 In Vitro Tumor‐Specific Targeting and Cell‐Killing

**Figure 2:**
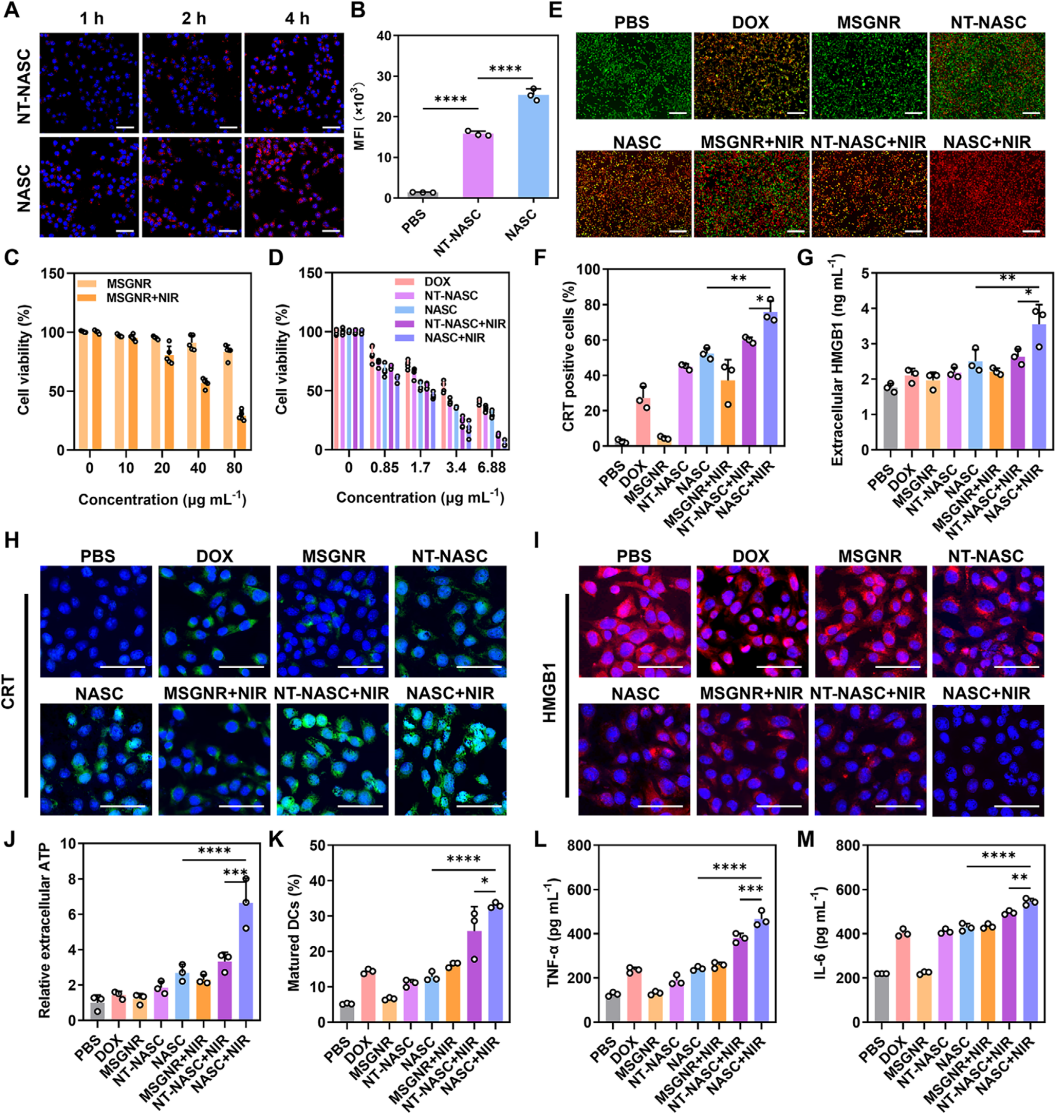
Cellular uptake and cytotoxicity of NASC nanorods in vitro. A) CLSM images of 4T1 tumor cells treated with NT‐NASC and NASC for 1, 2, and 4 h. Scale bar: 50 µm. B) Flow cytometric analysis of 4T1 tumor cells treated with NT‐NASC and NASC for 4 h. C) Cell viability of 4T1 tumor cells treated with MSGNR without or with laser irradiation. D) Cell viability of 4T1 tumor cells with different treatments. E) Live/dead imaging of 4T1 tumor cells under different treatments. Scale bar: 100 µm. F) Flow cytometry detection of CRT exposure on the cell surface of 4T1 tumor cells under different treatments. G) ELISA assay of extracellular HMGB1 content. Immunofluorescence staining of H) CRT and I) HMGB1. Scale bar: 50 µm. J) Extracellular ATP levels. K) The DCs maturation detected by flow cytometry. L) TNF‐α contents in the T cell culture medium. M) IL‐6 contents in the culture medium of T cells. Data were performed as the mean ± SD (*n* = 3). ^*^ *p* < 0.1, ^**^ *p* < 0.01, ^***^ *p* < 0.001, ^****^ *p* < 0.0001.

As the AS1411 aptamer has a high affinity for nucleoli that are usually overexpressed on the surface of tumor cells,^[^ ^25^ ^]^ AS1411‐bearing nanosystems will possess the ability to selectively target tumor cells. Therefore, the in vitro tumor‐targeted delivery efficiency was assessed on a breast carcinoma cell line (4T1 cells) by confocal laser scanning microscopy (CLSM) observation. To be specific, the AS1411 moiety in NASC was additionally replaced with a scrambled composition to construct the nontargeted control (designated as NT‐NASC). As observed in **Figure** **2**A, the red fluorescence signal of DOX in the 4T1 tumor cells treated with NASC was significantly brighter compared to the NT‐NASC group, indicating that abundant nanoparticles were internalized by tumor cells by endocytosis. Additionally, the fluorescence intensity was gradually increased with the increase of the incubation time, demonstrating that the drug‐loaded NASC can continuously enter tumor cells for intracellular drug delivery. In marked contrast, the nontargeted control nanosystem did not show significant cellular endocytosis even with a 4 h co‐incubation, which may be ascribed to the lack of targeting moiety in the nanosystem for tumor‐specific targeting. This targeting behavior was also demonstrated by flow cytometry analysis, leading to a ≈1.68‐fold higher increase in mean fluorescence intensity (MFI) in NASC‐treated cells versus NT‐NASC‐treated cells after 4 h incubation (Figure 2B). Moreover, as shown in Figure S2 (Supporting Information), quantitative ICP‐MS analysis revealed a time‐dependent enhancement in cellular uptake, with the Au accumulation in the NASC 4‐h treatment group reaching 4.71‐fold higher than that of the 1‐h group. Quantitative ICP‐MS analysis further revealed that NASC‐treated 4T1 cells exhibited significantly enhanced cellular uptake efficiency, with the 4‐h group demonstrating 75.98% Au internalization – a 1.34‐fold increase compared to NT‐NASC controls, further demonstrating the favorable tumor‐targeting efficacy of the designed NASC.

After effective cellular internalization by tumor cells, the NASC is expected to induce a synergistic cell‐killing effect via the combination of chemotherapy and PTT. To confirm this, the in vitro therapeutic effect of NACS was tested by cell counting kit‐8 (CCK8) assay. 4T1 tumor cells were co‐cultured with free DOX and nanoformulations including MSGNR, NT‐NASC, and NASC without or with 808 nm laser irradiation. As shown in Figure 2C, for 4T1 cells treated with bare MSGNR, the cell viability was barely influenced without laser irradiation (≈83.05% cell alive), whereas, with laser exposure, the cells were gradually killed with less than 27.01% cell survival at nanoparticle concentration of 80 µg mL^−1^, confirming the heat ablation effect of our designed nanosystem as an effective photothermal agent. For cells treated with DOX, a concentration‐dependent cytotoxic effect was achieved, which was increased when loading the drugs into nanocarriers (NT‐NASC treated group) (Figure 2D), owing to the efficient role of nanoparticulate systems in delivering drug molecules intracellularly to cause cell death. When endowing the nanoparticles with additional targeting ability (NASC treated group), the cell‐killing effect was further enhanced, leading to more than 71.5% of cells killed at the corresponding DOX concentration of 6.88 µg mL^−1^. Upon laser irradiation, the drug‐loaded formulation (NT‐NASC and NASC treated group) showed a much‐enhanced cell‐killing effect, especially for the targeted nanosystem (NASC+NIR treated group) that induced the maximum cytotoxicity (less than 5% cell survival ultimately). This was attributed to the additive cytotoxic effect of the delivered drug and in situ generated heat in synergistically inducing amplified cell death. Such an effective antitumor activity was further supported by live/dead cell staining to directly observe the cell‐killing effect by fluorescence microscope. As shown in Figure 2E, although free drugs or drugs loaded in nanosystems caused a death‐inducing capacity at varying degrees, these treatments were not efficient enough to kill tumor cells. The red fluorescence intensity indicating dead cells was significantly increased with concurrently decreased green fluorescence in NIR‐irradiated groups, compared with the corresponding nonirradiated groups (MSGNR vs MSGNR+NIR; NT‐NASC vs NT‐NASC+NIR; NASC vs NASC+NIR). The strongest red fluorescence with almost complete cell death was observed in the NASC+NIR group, suggesting that NASC endowing with AS1411 targeting moiety and loading DOX effectively targets and induces cell death in 4T1 tumor cells under NIR irradiation. These findings further emphasize the effective cell‐killing activity of the designed NASC against tumor cells.

### 2.3 In Vitro Induction of ICD and Immune Activation

Both anthracycline‐mediated chemotherapy and PTT can induce ICD. To confirm this, typical ICD markers including CRT, HMGB1, and ATP were evaluated under different treatments. By flow cytometry (Figure 2F; Figure S3, Supporting Information) and CLSM (Figure 2H; Figure S4A, Supporting Information) assay, the expression amount of CRT on the surface of 4T1 cells was significantly augmented by exposure to chemotherapeutic drug (DOX, NT‐NASC and NASC treated groups) and light‐induced heat (MSGNR, NT‐NASC and NASC treated groups under laser irradiation), as compared with the control and MSGNR groups. Notably, the synergism of chemo‐ and photothermal therapy (NASC+NIR treated group) showed the maximum CRT induction ability with more than 74.77% cells in a CRT‐positive rate, which exceeded other groups (PBS: 2.68%; DOX: 27.23%; MSGNR: 4.61%; NT‐NASC: 44.83%; NASC: 52.33%; MSGNR+NIR: 37.10%; NT‐NASC+NIR: 59.93%) considerably. By enzyme‐linked immune sorbent assay (ELISA) (Figure 2G) and CLSM observation (Figure 2I; Figure S4B, Supporting Information), an obviously high amount of HMGB1 (up to 2.63 ng mL^−1^) was released from 4T1 cells treated with NT‐NASC+NIR, which was much more than that in other control groups. Consistently, NASC+NIR treatment also increased the secretion of ATP from tumor cells, resulting in over 1.79‐fold higher than treatments including drug‐involved formulations (DOX, NT‐NASC, and NASC), the single heat stimulation (MSGNR+NIR), as well as the nontargeting formulation under laser irradiation (NT‐NASC+NIR) (Figure 2J). All these results strongly demonstrated the satisfied ICD‐induction ability of NASC under laser irradiation by the simultaneous effect of two ICD inducers (DOX and GNR photothermal agent) on tumor cells, which possessed great potential in evoking a robust immune response.

Regarding the above results, the significantly elevated levels of DMAPs (CRT, HMGB1, and ATP) from dying tumor cells were expected to stimulate the maturation of DCs, which was pivotal in the initiation of both innate and adaptive immunity. Therefore, bone marrow‐derived dendritic cells (BMDCs) co‐cultured with 4T1 tumor cells under different formulations of treatment were evaluated by flow cytometry analysis. The maturation rate occurred in the order as follows: PBS ≈ MSGNR < drug‐involved formulations (DOX, NT‐NASC, and NASC) < MSGNR+NIR < NT‐NASC+NIR < NASC+NIR, demonstrating the highest ability of NASC+NIR to mature BMDCs with a maturation rate of 32.9% (Figure 2K). Matured DCs were among the most effective means to activate T cells, which, in turn, were among the most potent immune cells to attack tumor cells for effective therapy. To assess the role of DCs in mediating T cell activation and cytokine production, splenic T cells were co‐incubated with the aforementioned BMDCs (Figure S5, Supporting Information). Furthermore, the levels of tumor necrosis factor α (TNF‐α) and interleukin‐6 (IL‐6) in the NASC+NIR group were significantly higher compared to other groups (Figure 2L,M). These results indicated that NASC exhibits strong tumor‐targeting ability and can effectively induce ICD under NIR treatment.

### 2.4 In Vivo Biodistribution of NASC

**Figure 3:**
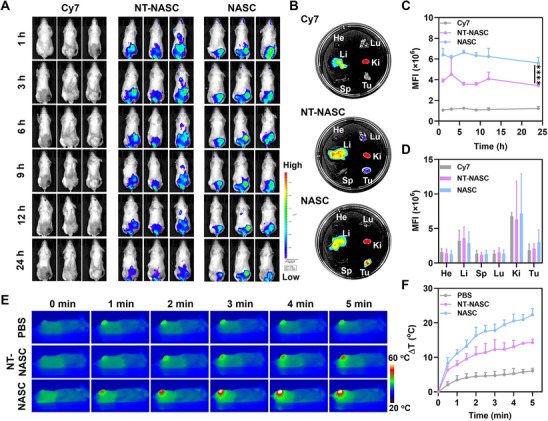
In vivo tumor targeting activity and photothermal effect of NASC. A) Whole‐body fluorescence imaging of 4T1 tumor‐bearing mice at designated time points post‐injection of Cy7, NT‐NASC, and NASC. B) Ex vivo fluorescence images of tumor, lung (Lu), liver (Li), kidney (Ki), spleen (Sp), and heart (He) after 24 h post‐injection. Quantitative evaluation of the mean fluorescence intensity (MFI) of C) tumor tissues at different time points and D) main organs/tumors after 24 h administration. E) Thermal images of mice treated with PBS, NT‐NASC, and NASC under laser irradiation (1.0 W cm^−2^, 5 min). F) Corresponding temperature change curves at local tumors. Data were performed as the mean ± SD (*n* = 3). ^****^ *p*< 0.0001.

To assess the AS1411 aptamer‐mediated tumor‐targeting property in vivo, the biodistribution of nanosystems (NT‐NASC and NASC) was investigated on mice bearing subcutaneous 4T1 tumor xenografts, in which the nanoparticles were labeled with Cy7 NIR probe for fluorescent observation. After intravenous injection, NASC‐treated mice revealed gradually increased fluorescence intensity at the tumor site, which reached the strongest at 6 h postinjection, and the fluorescence still remained at a high level even after 24 h injection (**Figure** **3**A). The modification of AS1411 aptamer would be responsible for such an excellent tumor targeting feature. As to NT‐NASC‐treated mice, much fewer NPs could enrich at the tumor site, and to Cy7‐treated mice, negligible fluorescence was detected. The ex vivo fluorescence of different tissues at 24 h postinjection also revealed that our designed nanocarrier displayed preferential tumor accumulation (Figure 3B), which was further confirmed by the quantitative evaluation of the tumor‐enriched fluorescence intensity at different time points (Figure 3C). Additionally, the biodistribution of nanosystems were evaluated in harvested tumors, along with key organs such as the heart, liver, spleen, lung, and kidney (Figure 3D). As a result, the targeted nanosystem of NASC exhibited superior tumor targeting compared to the nontargeted nanosystem of NT‐NASC, which would enable the highly efficient delivery of loaded cargo at diseased tumor sites.

Subsequently, the NIR laser‐triggered temperature change at the tumor site was performed on 4T1 tumor‐bearing mice. As real‐time monitored by an IR camera upon 808 nm laser irradiation (1 W cm^−2^, 5 min), the mice thermal image and the time‐dependent temperature at tumor tissue were displayed in Figure 3E,F. For the negative control group, little to no tumor temperature rise (ΔT < 6 °C) was observed, demonstrating that NIR irradiation alone without thermal agents cannot induce a significant change in living systems. For NT‐NASC‐treated mice, the local temperature of the tumors showed a moderate temperature increase (ΔT ≈ 14.3 °C) during the course of photo‐irradiation, which might be attributed to the inefficient accumulation of nontargeted nanosystems at the tumor region. For the mice treated with NASC at the same laser power density, a faster temperature increase rate was detected, and the ultimate temperature in tumor tissue reached ≈56.7 °C after 5 min irradiation, which was sufficient enough to cause hyperthermic cell damage in PTT.

### 2.5 Antitumor Immune Response Evoked by NASC

**Figure 4:**
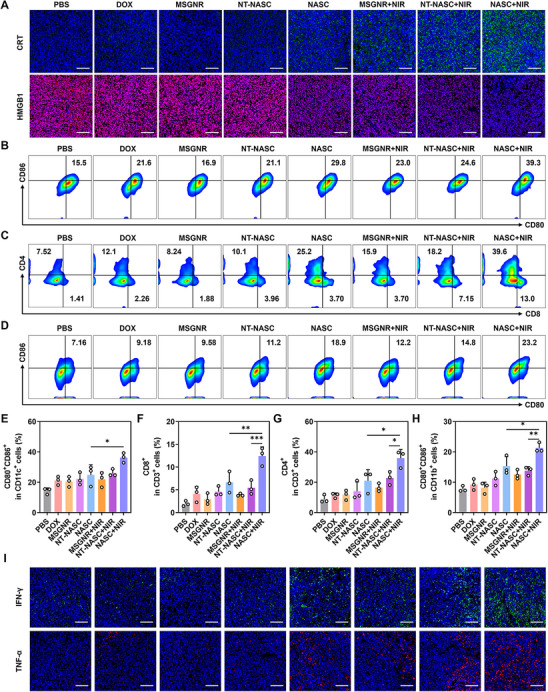
Antitumor immune responses evoked by NASC+NIR treatment. A) Immunofluorescence staining of CRT and HMGB1 in tumor sites from unilateral 4T1 tumor‐bearing mice. Scale bar: 100 µm. Flow cytometry analysis of B) DCs, C) T cells, and D) M1 macrophages in tumor sites from unilateral 4T1 tumor‐bearing mice under different treatments. E‐H) The corresponding quantitative analysis. I) Immunofluorescence images of TNF‐α and IFN‐γ contents in unilateral tumor sections. Scale bar: 100 µm. Data were performed as the mean ± SD (*n* = 3). ^*^ *p* < 0.1, ^**^ *p* < 0.01, ^***^ *p* < 0.001.

After evaluating the biological distribution of NASC NPs, we next investigated their ability to induce antitumor immunity in a 4T1 tumor‐bearing mouse model. The release of HMGB1 and CRT was used to assess the levels of DAMPs in the tumor microenvironment (TME) (**Figure** **4**A). Notably, fluorescence imaging of the NASC‐treated group revealed strong signals of CRT and weak signals of HMGB1, while the PBS group showed only faint fluorescence of CRT and intense fluorescence of HMGB1, indicating that NASC NPs could induce DAMPs release. Furthermore, the NASC+NIR group exhibited even stronger fluorescence signals of CRT and weaker signals of HMGB1 than the NASC‐only group, suggesting that the photothermal effect of NASC NPs enhanced DAMP release, which was beneficial for following activation of antitumor immune responses.

Encouraged by the massive DAMPs release induced by NASC+NIR treatment in the TME, the infiltration of immune cells was further evaluated. To investigate the inflammatory response and the activation of dendritic cells in tumors, NASC nanoparticles were administered into tumor‐bearing mice via tail vein injection, and irradiated with 5‐min near‐infrared post‐injection. After 7 days, the tumor tissues were collected to evaluate dendritic cell maturation by flow cytometric analysis. The maturation of DCs was significantly higher in the NASC‐treated group, reaching 24.8%, which was ≈1.73 times greater than that observed in the PBS group (14.3%) (Figure 4B,E). This result demonstrated that NASC treatment effectively enhanced DC maturation by inducing moderate DAMPs release. Notably, laser irradiation further boosted DC maturation in the NASC+NIR group (36.3%) (*p* < 0.001), which was 1.46 times greater than the NASC group and 1.39 times greater than the NT‐NASC+NIR group (26.0%). These results demonstrated that NASC with the enhanced targeting ability had the excellent ability to induce ICD under irradiation, which supported the effective maturation of DCs. In addition, the frequency of CD3^+^CD8^+^ T cells in the NASC+NIR group reached 12.4%, compared to only 1.94% in the control group, with a similar trend observed in tumor‐infiltrating CD3^+^CD4^+^ T cells (Figure 4C,F,G). These findings suggested that NASC+NIR treatment effectively enhanced the abundance of T lymphocytes within the TME. Furthermore, the presence of M1 macrophages in the NASC+NIR group significantly increased, indicating NASC+NIR treatment could mediate the polarization of macrophages to proinflammatory M1 type (Figure 4D,H). In Figure 4I, Immuno‐fluorescence staining of TNF‐α and IFN‐γ at tumor sites also showed a significant trend, in which signals of groups with NIR were much stronger than groups treated with the same nanoparticles but without NIR, and the strongest signals observed in the NASC+NIR group. Also, the levels of tumor necrosis factor IL‐6 in the NASC+NIR group were notably higher compared to other groups in blood content (Figure S6, Supporting Information). Collectively, these results indicated that NASC+NIR treatment could evoke robust immune responses by inducing the ICD of tumor cells within the TME.

### 2.6 In Vivo Tumor Therapy

**Figure 5:**
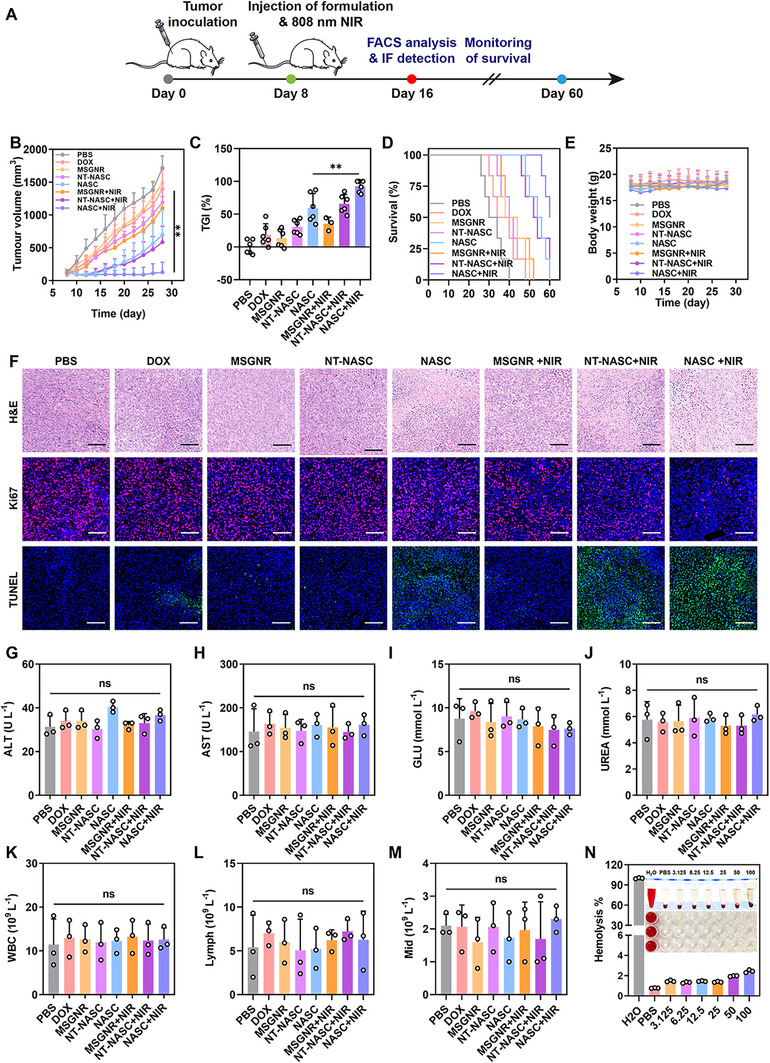
In vivo antitumor effect of NASC+NIR on unilateral 4T1 tumor‐bearing mice. A) Schematic diagram of the treatment schedule. B) Tumor growth curves, C) tumor growth inhibition (TGI) values, D) survival rates, and E) mice body weight changes of 4T1 tumor‐bearing mice in different groups. F) H&E, Ki67, and TUNEL staining of the harvested tumors. Scale bar: 100 µm. G–M) Analysis of the blood biochemistry and blood routine indexes of mice with different treatments at day 14. N) The hemolysis rates of mouse blood after co‐incubation with NASC in different concentrations. Data are represented as mean values ± SD. *n* = 6 biologically independent samples in Figure B‐E. *n* = 3 biologically independent samples in Figure G‐N. ^**^ *p* < 0.01.

Based on effective extracorporeal cell killing, tumor‐homing, and immune activation features of NASC, the combination antitumor activity of chemotherapy, PTT, and immunotherapy was investigated on mice bearing 4T1 tumors. When the tumor volume reached ≈100 mm^3^, the mice were randomly divided into eight treatment groups, which dealt with treatments including PBS, DOX, MSGNR, NT‐NASC, NASC, MSGNR+NIR, NT‐NASC+NIR, and NASC+NIR (*n* = 6) (**Figure** **5**A). From the tumor growth curves in Figure 5B and Figure S7 (Supporting Information), tumors in the PBS group grew unpredictably fast, with the average tumor size increasing by 10.24‐fold on day 26 compared to the tumor size on the day of administration. A negligible antitumor effect was observed in the MSGNR‐treated mice. For single chemotherapy, only weak or mild tumor suppression was detected, revealing an 8.43‐ (DOX treatment), 8.82‐ (NT‐NASC treatment), and 5.16‐fold (NASC treatment) increase of the tumor volume, respectively, and the tumor suppression activity of DOX was promoted via delivering using nanosystems, especially with targeting ligand modification. For a single PTT treatment (MSGNR+NIR group), the tumor growth could be inhibited to a certain extent. By additional laser irradiation of NT‐NASC and NASC groups to combine the DOX‐mediated chemotherapy, thermal ablation effect, and the potential immunotherapeutic role, extraordinary tumor regression was achieved, especially in the NASC+NIR group owing to its active targeting ability to tumors. Calculated from the tumor growth volumes, NASC+NIR group revealed the highest tumor growth inhibition (TGI) value (92.54%) in comparison with NASC group (59.3%) and NT‐NASC+NIR group (65.9%) (Figure 5C). In virtue of the superior antitumor efficacy, the NASC+NIR group demonstrated the longest survival time and the highest survival rate (Figure 5D). Notably, the weight of mice in all groups remained stable with no abnormal fluctuation during the whole treatment process (Figure 5E). Moreover, histological analysis using hematoxylin and eosin (H&E) staining, along with Ki‐67 and in situ terminal deoxynucleotidyl transferase dUTP nick end labeling (TUNEL) staining at the tumor sites, further confirmed the robust antitumor effect of NASC+NIR, as revealed by massive cell damage and death (Figure 5F).

Moreover, the hematological parameters, including aminotransferase (ALT), aspartate aminotransferase (AST), glucose (GLU), blood urea nitrogen (UREA), white blood cell (WBC), lymphocyte (lymph) and median cell (Mid) across all groups fell within normal ranges (Figure 5G–M), demonstrating that almost no damage occurred to influence the renal and hepatic functions. Additionally, H&E staining of tissue sections from the heart, liver, spleen, lung, and kidney of the treated mice revealed no noticeable inflammation lesions and pathological damages in all groups (Figure S8, Supporting Information). The hemolysis assay shown in Figure 5N indicated negligible hemolytic activity of all the nanoformulations (percent hemolysis < 4%). All these results revealed the good biosafety of NASC and its control formulations, which was critically important for usage in biomedical applications.

### 2.7 Antitumor Effects Arbitrated by NASC+NIR Connected with Immune Checkpoint Blockade

**Figure 6:**
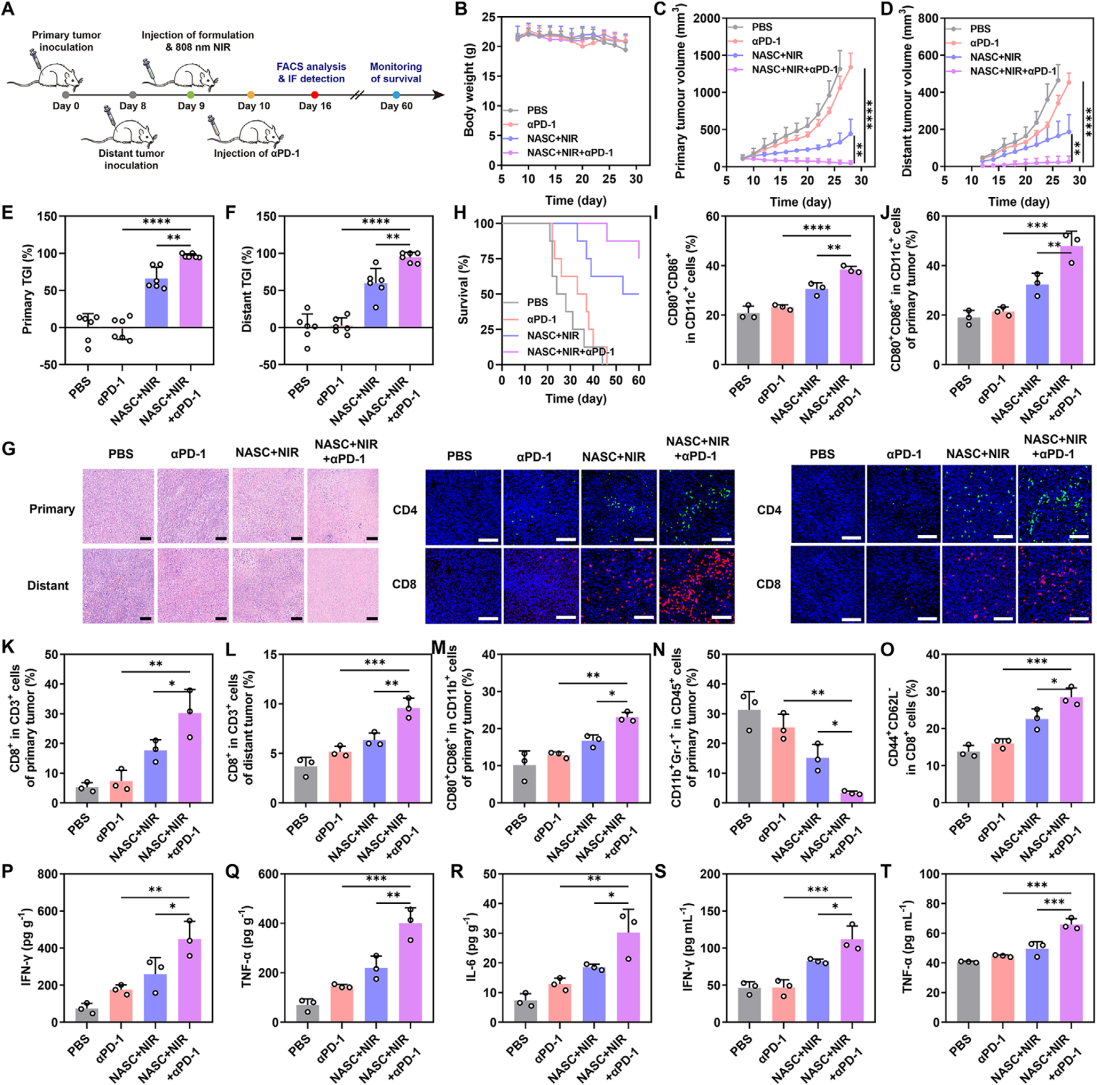
Antitumor activity of NASC+NIR+αPD‐1 treatment on 4T1 bilateral tumor models. A) Schematic diagram of the treatment schedule. B) Mice body weight changes, C) primary tumor growth curves, D) distant tumor growth curves, E) primary TGI, F) distant TGI, G) survival rates of bilateral 4T1 tumor‐bearing mice in different groups. H) DCs maturation in lymph nodes. I) DCs maturation in primary tumors. CD3^+^CD8^+^ T cells in J) primary tumors and K) distant tumors. L) M1 macrophages and M) MDSC at primary tumor sites detected by flow cytometry. N) Flow cytometry analysis of effector memory T cells in spleens. O) Intratumoral contents of O) IFN‐γ, P) TNF‐α, and Q) IL‐6 measured by ELISA assay. Blood contents of R) IFN‐γ and S) TNF‐α. T) Immunofluorescence images of H&E, CD4, and CD8 in bilateral tumor sections. Scale bar: 100 µm. Data are represented as mean values ± SD. *n* = 6 biologically independent samples in Figure B–F. *n* = 3 biologically independent samples in Figure I–T. ^*^ *p* < 0.1, ^**^ *p* < 0.01, ^***^ *p* < 0.001, ^****^ *p* < 0.0001.

Given that NASC+NIR treatment can achieve satisfactory therapeutic effects, we further explore the systemic antitumor effects after combining with immune checkpoint blockade (ICB) in 4T1 bilateral tumor models (**Figure** **6**A). The treatment of NASC along with anti‐programmed cell death protein 1 antibody (αPD‐1) was administered to 4T1 bilateral tumor‐bearing mice. Among these groups, the changes in mouse weight were negligible during treatment, further confirming the biosafety of the treatment (Figure 6B). For the curves of tumor volumes in the primary and distant sites, the PBS group and the αPD‐1 group had similar tumor growth rates, indicating that ICB treatment alone could not achieve satisfactory therapeutic effects (Figure 6C,D; Figure S9, Supporting Information). Notably, the bilateral tumor volumes significantly decreased after NASC+NIR+αPD‐1 treatment. The TGI for the NASC+NIR+αPD‐1 group in primary tumor and distant tumor was 96.55% and 94.54%, while the NASC+NIR group showed a TGI of only 66.3% and 59.72%, indicating that the tumor‐targeting NASC under NIR effectively suppressed bilateral tumor growth when combined with anti‐PD‐L1 antibodies (Figure 6E,F). Meanwhile, H&E staining of bilateral tumor tissues revealed that the NASC+NIR+αPD‐1 treatment exhibited the most effective tumor cell‐killing ability among all the treatment groups, highlighting its excellent synergistic antitumor effects (Figure 6G). Furthermore, the survival time of the tumor‐bearing mice was significantly prolonged after the combination therapy, indicating a significant improvement in therapeutic effects (Figure 6H). In general, these results illustrated that the combination of chemotherapy and PTT mediated by NASC+NIR treatment to induce immune responses enhances the effectiveness of ICB therapy, leading to more powerful tumor suppression.

Subsequently, we explored the mechanism by which the NASC+NIR+αPD‐1 combination treatment inhibited distant tumor growth. From the analysis of flow cytometry, NASC under NIR significantly enhanced the ICD effect, increasing the proportion of mature DCs in the lymph nodes from 24.1% to 37.8% (Figure 6I; Figure S10, Supporting Information). Furthermore, DCs maturation in primary tumors in the NASC+NIR+αPD‐1 group (47.97%) was also significantly higher than the NASC+NIR group (32.43%) (Figure 6J; Figure S11, Supporting Information). Additionally, the highest proportion of CD3^+^CD8^+^ T cells in primary tumors was observed in the NASC+NIR+αPD‐1 group (30.8%), significantly higher than that in the NASC+NIR group (Figure 6K; Figure S12, Supporting Information). Notably, CD3^+^CD8^+^ T cells in distant tumors also increased considerably in the NASC+NIR+αPD‐1 group compared to the PBS group and the αPD‐1 group (Figure 6L; Figure S13, Supporting Information), indicating combination treatment could activate a systemic immune response and further enhance the infiltration of T cells to distant tumors. Immunofluorescence staining of CD4^+^ and CD8^+^ at tumor sites also shows a similar trend, in which the strongest signals were observed in the NASC+NIR+αPD‐1 group (Figure 6G). Notably, the number of M1 macrophages in primary tumors treated with NASC+NIR+αPD‐1 was significantly higher compared to the other groups (Figure 6M; Figure S14, Supporting Information). Meanwhile, the lowest proportion of myeloid‐derived suppressor cells (MDSCs) in primary tumors was found in the NASC+NIR+αPD‐1 group (3.18%) compared to the other groups, indicating that this treatment can effectively induce an immune response without triggering MDSC‐mediated immune suppression (Figure 6N; Figure S15, Supporting Information). The proportion of effector memory T cells in the spleen was also significantly elevated in the NASC+NIR+αPD‐1 group (Figure 6O; Figure S16, Supporting Information). Moreover, we analyzed cytokine levels in blood and tumor sites using ELISA assays. The levels of TNF‐α, IFN‐γ, and IL‐6 in the NASC+NIR+αPD‐1 group were significantly elevated compared to the other groups (Figure 6P–T). These results suggest NASC+NIR+αPD‐1 treatment can evoke robust systemic immune responses for bilateral tumor growth inhibition.

## 3 Conclusion

In summary, an “all‐in‐one” smart carrier, NASC, aiming at combining multiple therapeutic modalities was constructed by locking the MSGNR‐based drug reservoir with the bioactive NAs. NASC was demonstrated to specifically target malignant cells through the receptor recognition function of AS1411 aptamer, showing abundant nanoparticles existed in vitro tumor cells and at local tumor tissues in vivo. Upon intracellular endocytosis, the ATP biomarker in tumor cells triggered the release of encapsulated DOX, which could not only elicit direct cell killing but also trigger ICD. In combination with GNR‐mediated PTT, this smart carrier could effectively destroy tumor cells more efficiently and further amplify ICD, resulting in massive DAMPs release to promote the infiltration of immune cells, reprogram the immunosuppressive TME, and ultimately boost antitumor immunity. With additional αPD‐1‐mediated immunotherapy, a robust antitumor activity was achieved, showing significant tumor growth suppression on both primary and distant tumor models. As designed, NASC‐mediated the combination of photothermal therapy, chemotherapy, and immunotherapy showed the complementary mechanism and synergistic advantages, such as PTT‐enhanced chemotherapy, PTT‐ and chemotherapy‐stimulated antitumor immunotherapy, which collectively potentiated the therapeutic effects to eliminate tumors. Overall, this work provides a promising tumor‐targeted ICD amplifier for enhancing the effectiveness of cancer therapy.

## 4 Experimental Section

### Controlled Drug Release

Briefly, 20 mg of NASC was dispersed in 4 mL PBS buffer solution, which was divided into four parts evenly and put into dialysis bags (MWCO: 3500 Da). The drug release performance was carried out under four different conditions: 1) without any treatment; 2) irradiation with 808 nm laser for 10 min (power: 1.0 W cm^−2^); 3) addition of 20 mm of ATP; 4) addition of 20 mm of ATP and irradiation with 808 nm laser for 10 min (power: 1.0 W cm^−2^). 3 parallel samples were included in per group. The dialysates were collected at specific time intervals, and the fluorescence spectrophotometer (RF5301 PC Spectro fluorophotometer) was used to measure the released DOX.

### Statistical Analysis

Statistical analysis was performed using GraphPad Prism 8.1. Data are presented as the mean values ± standard deviation (SD). One‐way ANOVA with Tukey's multiple comparisons test was employed for the statistical analysis of multiple groups. A significance threshold of *p <* 0.05 was considered statistically significant for all tests, ^⁎^ *p <* 0.05, ^⁎⁎^ *p <* 0.01, ^⁎⁎⁎^ *p <* 0.001, ^⁎⁎⁎⁎^ *p <* 0.0001, and ns denotes no significant difference.

## Conflict of Interest

The authors declare no conflict of interest.

## Supporting information



Supporting Information

## Data Availability

The data that support the findings of this study are available from the corresponding author upon reasonable request.
