# Supplementary material for: Nucleic Acid‐Locked Smart Carrier for Photothermal/Chemotherapy‐Amplified Immunogenic Cell Death to Enhance Systemic Antitumor Efficacy
Source: Adv Sci (Weinh). 2025 Apr 4;12(26):2503299. doi: 10.1002/advs.202503299 (PMC12245015; doi:10.1002/advs.202503299)
Supplement: Supplementary file 1 — Supporting Information [file ADVS-12-2503299-s001.docx]

Supporting Information for

Nucleic Acid-Locked Smart Carrier for Photothermal/Chemotherapy-Amplified Immunogenic Cell Death to Enhance Systemic Antitumor Efficacy

Jiayang Zhang,^a^ Tianyu Gai,^a^ Jiwei Wang, Yucai Wu,^a^ Si-Ming Zeng,^b^ Dongyuan Zhao,^a,*^ and Wei Li,^a,*^

^a^ Department of Chemistry, Laboratory of Advanced Materials, Shanghai Key Laboratory of Molecular Catalysis and Innovative Materials, Fudan University, Shanghai 200433, P. R. China

^b^ Key Laboratory of Biomedical Polymers of Ministry of Education & Department of Chemistry, Wuhan University, Wuhan 430072, P. R. China

**Materials and methods**

**Reagents and Medicines.** Tetra chloroauric acid (HAuCl_4_•3H_2_O), sodium borohydride (NaBH_4_), silver nitrate, and ascorbic acid (AA) were purchased from Sigma-Aldrich. Tetraethyl orthosilicate (TEOS), CTAB, 3-aminopropyltriethoxysilane (APTES), succinic anhydride, N-hydroxy succinimide (NHS), and 1-(3-dimethylaminopropyl)-3-ethylcarbodiimide hydrochloride (EDC•HCl) were obtained from Shanghai Chemical Co. (Shanghai, China) and used directly. All DNA sequences were synthesized by Sangon Biotech Co., Ltd. (Shanghai). Dimethyl sulfoxide (DMSO), and methanol (MeOH) were purchased from Shanghai Chemical Co. (Shanghai, China). Doxorubicin hydrochloride (DOX•HCl) was obtained from Zhejiang Hisun Pharmaceutical Co., Ltd. Fetal bovine serum (FBS), trypsin, penicillin-streptomycin, Dulbecco’s PBS, and nuclear staining dye (Hoechst 33342) were purchased from Invitrogen (USA). αPD-1 was acquired from InVivoMAb, Inc (USA). Roswell Park Memorial Institute 1640 (RPMI-1640) was purchased from Invitrogen.

**Characterization.** The morphology and particle size of gold nanorods and mesoporous silica-coated gold nanorods were characterized by transmission electron microscope (TEM, JEM-F200, JEOL Ltd, Japan). The pore size distribution, surface area, and pore volume were characterized by a porosity Analyzer (BET, BJH, ASAP 2020, Micromeritics). The hydrodynamic size and zeta potential of different nanoparticles were assessed by dynamic light scattering (DLS, Malvern Zetasizer ZEN3600). The absorption spectra of different nanomaterials were measured by an ultraviolet-visible spectrophotometer (LAMBDA Bio40, PerkinElmer). An 808 nm near-infrared (NIR) laser was used for photothermal performance conversion and photothermal treatment research (Hi-Tech Optoelectronics Co., Ltd, LOS-BLD-0808-005W). The light-to-heat conversion and near-infrared imaging were detected by a thermal camera (JIR-A384). A BD Accouri C6 flow cytometer was used in flow cytometric analysis. IVIS imaging system (PerkinElmer) was used in in vivo imaging experiments. Blood Biochemistry Analyzer (MNCHIP POINT CARE) and Auto Hematology Analyzer (MC-6200VET) were used to analyze blood biochemistry and blood routine.

**Cell Culture.** The 4T1 tumor cells were obtained from the China Center for Type Culture Collection (CCTCC) and grown in RPMI-1640, containing 10% fetal bovine serum (FBS, VivaCell), 1% penicillin-streptomycin solution (SP002030100, Sperikon Life Science & Biotechnology co., ltd) in an incubator at 37 °C with 5% CO_2_.

**Synthesis of MSGNR.** First, the gold nanorods were prepared by the seed growth method. Briefly, 2.0 mL of HAuCl_4_ (0.5 mM) solution was mixed with 2.0 mL of CTAB solution (0.20 M). Then, 0.24 mL NaBH_4_ (0.01 M) cold solution was added to the above mixture under constant stirring. The resultant reaction solution immediately turned brownish yellow. The reaction solution was vigorously stirred for 2 min and then stored at 25 °C to obtain the seed solution (note: use within 3 h).

The growth solution of gold nanorods was prepared as follows: 200 mL of CTAB (0.2 M), 11.2 mL of AgNO_3_ (4 mM), 13 mL of HAuCl_4_ (23 mM), and 190 mL of ultrapure water were mixed well and stirred at a constant speed. Then, 5 mL of AA (0.08 M) was slowly dropped into the solution, which turned from brownish-yellow to colorless. Thereafter, 3.6 mL of seed solution was added to the growth solution, and the reaction was continued for 12 h at room temperature. The obtained gold nanorods were washed with a large amount of ultrapure water and centrifuged (11000 rpm, 25 min) to remove the excess CTAB. The resulting precipitate was dispersed in 100 ml ultrapure water, in which 1 mL of NaOH (0.1 M) solution was added. Finally, 100 µL of TEOS/MeOH (20%, V/V) mixture was added to the reaction solution every 30 min for a total of 3 times. The MSGNR was obtained after 6 h of mild stirring and the reaction was washed twice with water to remove the unreacted substances.

**Synthesis of MSGNR-NH_2_.** 3 mL of APTES was added to 50 mL of anhydrous MeOH solution containing MSGNR (200 mg), which was continually stirred for 5 h at room temperature. Afterward, the resultant nanoparticles were refluxed in HCl/MeOH (1%, V/V) solution for 6 h to remove CTAB and subsequently washed with a large amount of MeOH thoroughly. Then, the nanoparticles were collected via centrifugation and dried in a vacuum to obtain MSGNR-NH_2_.

**Synthesis of MSGNR-acDNA.** Anchoring DNA (150 µL, 0.1 mmol), NHS (200 µL, 10 mg/mL), and 87.4 mg EDC·HCl (200 µL, 10 mg mL^-1^) were mixed in 1 mL of ultrapure water at room temperature. The reaction was stirred for 1 h to activate the carboxyl group on the DNA sequence. Then, 1 mg MSGNR-NH_2_ nanoparticles were added to the above-mixed solution and continued to react for another 6 h. After centrifugation and washing with PBS several times, MSGNR-acDNA was obtained.

**Synthesis of NASC.** Briefly, 2.0 mg of MSGNR-acDNA was dispersed in a DOX (1 mg mL^-1^) PBS buffer solution (pH 7.4, 10 mM) and oscillated overnight at room temperature. Then, the drug loaded nanoparticles were incubated in a AS1411/ATP aptamer DNA (200 µL, 0.1 mmol) solution under stirring for 6 h at room temperature, resulting in the formation of drug-loaded, nucleic acid-locked, NASC nanoparticles. After centrifugation and washing with PBS several times, NASC nanoparticles were obtained. The loading content of DOX was determined by the established fluorescence standard curve.

**Photothermal experiment in vitro.** Prepared NASC with different concentrations were placed in EP tubes. Irradiated by an 808 nm laser (1.0 W cm^-2^) for 7 minutes. The solution temperature was recorded every 30 seconds and a near-infrared thermal image was taken by a photothermal imager every 1 minute. Also, NASC at a fixed concentration of 20 µg mL^-1^ was irradiated by different laser powers (0.5 W cm^-2^, 1.0 W cm^-2^, 1.5 W cm^-2^) for 7 minutes. The solution temperature was recorded every 30 seconds and a near-infrared thermal image was taken by a photothermal imager every 1 minute.

**Cellular Uptake Analysis.** 4T1 tumor cells were cultured overnight in cell dishes. The next day, Cy5.5-labeled NT-NASC (2mg mL^-1^) and NASC (2mg mL^-1^) were co-incubated with the 4T1 tumor cells. After incubation for 1 hour, 2 hours, and 4 hours, the cells were washed twice with PBS and analyzed using confocal laser scanning microscopy (CLSM) and flow cytometry (BD Accuri C6, USA).

**Cytotoxicity Test.** Initially, 4T1 cells were plated in a 96-well culture plate at a density of 1×10^5^ cells per well. The cells were then incubated for 24 hours. Following this, different concentrations of MSGNR were dispersed in 100 μL of RPMI-1640 medium with 10% FBS and 1% antibiotics, and the mixture was co-cultured with the cells at 37 °C for 4 hours. After this, the cells were washed three times with PBS, and a fresh RPMI-1640 medium was added. To investigate the effect of NIR laser, the co-cultured cells with the nanocomposites were exposed to an 808 nm laser for 3 minutes at a power density of 1.0 W cm^-2^, and then the cells were further cultured for 8 hours. After this, a culture medium containing 10% CCK-8 solution was added. The cells were then incubated for 2 hours, and the absorbance of the culture medium at 450 nm was measured using a microplate reader. This procedure was also used to evaluate the effects of various conditions, including different concentrations of DOX, NT-NASC, NT-NASC+NIR, NASC, and NASC+NIR groups.

**ICP Analysis.** Mixed 2-3 mL of cell suspension with 24 mL complete medium. And seeded three 6-well plates (2 mL/well). After about 24 hours, replaced medium with fresh medium (2 mL/well). And then added NT-NASC NPs and NASC NPs (NASC: 100μg/well; 2mg mL^-1^) for 1h, 2h, 4h, respectively. Aspirated medium carefully, then washed gently with PBS (2-3 times). Digested cells with 0.25% trypsin-EDTA and neutralize digestion with complete medium. Finally, transferred cell suspension to 1.5 mL centrifuge tube. And centrifuge at 3000 × g for 5 min. Next, resuspended cell pellet in 200 μL concentrated HNO₃ and digested overnight at room temperature. The next day, added 800 μL of ultrapure water, mixed well, and filtered the solution through a 0.45 μm filter into a new tube. And finally, stored samples in labeled tubes for ICP analysis. ICP analysis was tested from the Core Facility of Wuhan University.

**Live/Dead cell staining assay.** The viability of 4T1 tumor cells was assessed using a Live/Dead cell staining assay. The 4T1 tumor cells were incubated with DOX (100μg/well; 1mg mL^-1^), MSGNR (100μg/well; 2mg mL^-1^), MSGNR+NIR (100μg/well; 2mg mL^-1^), NT-NACS (100μg/well; 2mg mL^-1^), NT-NACS+NIR (100μg/well; 2mg mL^-1^), NACS (100μg/well; 2mg mL^-1^), or NACS+NIR (100μg/well; 2mg mL^-1^) for 12 h at 37 °C, respectively, then irradiated by 808 nm laser for 3 minutes at a power density of 1.0 W cm^-2^, and co-cultured for 8 h. After staining using fda and pi, the cells were washed three times with PBS and examined under an inverted fluorescence microscope.

**Assessment of Anti-Tumor Immune Responses Triggered by NASC in vitro.** The release of HMGB1 in 4T1 cells was measured using an ELISA kit（Solarbio）and CLSM. At the same time, calreticulin (CRT) expression was assessed through immunofluorescence analysis using a flow cytometer and CLSM. The cells were cultured in 6-well plates overnight at 37 °C, then treated with DOX (100μg/well; 1mg mL^-1^), MSGNR (100μg/well; 2mg mL^-1^), MSGNR+NIR (100μg/well; 2mg mL^-1^), NT-NACS (100μg/well; 2mg mL^-1^), NT-NACS+NIR (100μg/well; 2mg mL^-1^), NACS (100μg/well; 2mg mL^-1^), or NACS+NIR (100μg/well; 2mg mL^-1^) for 4 h, respectively. After this, the cells were washed three times with PBS, then a fresh RPMI-1640 medium was added, and the 4T1 tumor cells were treated with an 808 nm laser (1.0 W cm^-2^, 3 minutes), then, co-cultured for 8 h. Afterward, the culture supernatant was collected for HMGB1 detection. Finally, the cells were analyzed with CRT or HMGB1 antibodies using either a flow cytometer or CLSM.

**In Vitro BMDCs Activation*.*** Bone marrow-derived dendritic cells (BMDCs) were isolated from the bone marrow of BALB/c mice, and single-cell suspensions were plated in culture dishes with RPMI-1640 medium containing IL-4 (10 ng mL^-1^) and murine GM-CSF (20 ng mL^-1^). Every two days, the culture medium was replaced with a fresh medium. On day 8, immature BMDCs, including both non-adherent and loosely adherent cells, were harvested for further experiments. Next, BMDCs (1×10^6^ cells per well) were seeded in 6-well plates. 4T1 cells (5×10^4^ cells per well) were seeded in 24-well plates and incubated for 24 hours. The 4T1 tumor cells were then treated with PBS, DOX, MSGNR, MSGNR+NIR, NT-NACS, NT-NACS+NIR, NACS, or NACS+NIR (NACS: 2mg mL^-1^) for 4 hours. The cells were washed three times with PBS, then a fresh RPMI-1640 medium was added, and the 4T1 tumor cells were treated with 808 nm laser (1.0 W cm^-2^, 3 minutes), the cells were then incubated for an additional 8 hours. Next, the supernatants were collected and transferred to BMDCs. After 24 hours, the BMDCs were harvested and stained with anti-CD11c-FITC, anti-CD80-PE, and anti-CD86-APC antibodies purchased from BioLegend for flow cytometry analysis. The supernatants were also assayed using ELISA kits for TNF-α, and IL-6.

**Animals and Tumor Models.** All animal experiments were conducted at the Animal Biosafety Level III Laboratory of Wuhan University. All procedures were carried out following the Regulations for the Administration of Affairs Concerning Experimental Animals, as approved by the State Council of the People's Republic of China. The Institutional Animal Care and Use Committee (IACUC) at Wuhan University's Animal Experiment Center (Wuhan, China) authorized all animal research (WP20240080). Subcutaneous 4T1 tumor models were developed using female BALB/c mice aged 6-8 weeks. To establish the 4T1 tumor-bearing mouse model, 100 μL of 4T1 cell suspension (1×10^7^ cells mL^-1^) was subcutaneously injected into the flank of BALB/c mice. The permission number of animal experiment is WP20240080.

**Imaging In Vivo.** Tumor-bearing mice were divided into three groups when tumor volume reached 150 mm^3^. Cy7-labeled NT-NASC, Cy7-labeled NASC, and Cy7 were injected into the tumor-bearing mice through the tail vein. Next, the mice were anesthetized at the designed time points (1 h, 3 h, 6 h, 9 h, 12 h, and 24 h) and imaged by using the IVIS imaging system. After 24 hours, all mice were sacrificed, then the major organs (heart, liver, spleen, lung, and kidney) and tumors were taken out and washed by PBS and imaged by using the IVIS imaging system.

**Photothermal Experiment In Vivo.** Prepared similar weights of 4T1 tumor-bearing mice and randomly divided into 3 groups. Prepared NASC NPs, NT-NASC NPs, and PBS to inject into the tail veil (NT-NASC and NASC: 2 mg mouse^-1^). After injection for 6 h, the tumor site of mice was irradiated by an 808 nm laser (1.0 W cm^-2^) for 7 minutes. The solution temperature was recorded every 1 minute and a near-infrared thermal image was taken by a photothermal imager every 1 minute.

**Antitumor Study In Vivo.** When the tumor volume reached about 100 mm^3^, the tumor-bearing mice were randomly divided into eight groups with 6 mice in each group, and received the following treatments: 1) PBS, 2) DOX, 3) MSGNR, 4) NT-NASC, 5) NASC, 6) MSGNR+NIR, 7) NT-NASC+NIR, 8) NASC+NIR (corresponding DOX concentration: 1 mg kg^-1^; laser irradiation: 3 mins, 1.0 W cm^-2^). The weight of the mouse was measured with a balance, and the size of the tumor was measured with a vernier caliper every two days. The tumor volume was calculated by the following formula: V = W^2^×L/2, where W and L represent the shortest and longest diameters of the tumor, respectively. On the 20th day of treatment, all mice were sacrificed and the experiment was ended. Subsequently, the major organs of the mice were examined histologically, and the tumors were analyzed using hematoxylin-eosin (H&E), Ki67, IFNγ, and TNFα staining. Blood samples were collected on day 16 for blood biochemical analysis and routine blood tests.

**Antitumor Immune Responses In Vivo.** In addition, immune responses were assessed via flow cytometry and ELISA assay kit from the Center of Biological Technology of Yuanji in Shanghai. Lymph nodes were harvested for analysis of dendritic cell (DC) maturation. Tumors were enzymatically dissociated in a solution containing 1 mg mL^-1^ collagenase, 0.1 mg mL^-1^ hyaluronidase, and 0.2 mg mL^-1^ DNase I to generate a single-cell suspension. This suspension was used to evaluate the frequencies of tumor-infiltrating lymphocytes through flow cytometry. Cytokine levels (IL6) in blood content were measured by ELISA.

**Therapeutic Effect of NASC Combined with Immune Checkpoint Blocking Treatment.** 32 tumor-bearing mice were randomly divided into four groups when tumor volume reached 100 mm^3^, including PBS, αPD-1, NASC+NIR, NASC+NIR+αPD-1, then received inject on the tail vein with the concentration of nanorods was 100 μL (20 mg mL^-1^) per mouse, and αPD-1 antibody was (20 μg per mouse, Clone: 29F.1A12, Biolegend) injected one day later. On day 16, lymph nodes were harvested to detect matured dendritic cells (DCs), and intratumorally immune responses were assessed by flow cytometry and ELISA assay. For flow cytometry, the primary tumors, spleens, and lymph nodes were obtained. To evaluate the DC maturation (CD11c^+^CD80^+^CD86^+^), the lymph nodes in mice were collected to obtain lymphocytes, and the lymphocytes were stained with anti-FITC-CD11c, anti-PE-CD80, and anti-APC-CD86 antibodies. The primary tumors of mice also were collected to obtain DC maturation. And antibodies used were as same as above. For analyzing the infiltration of CD8^+^ T cells (CD3^+^ CD8^+^), M1 macrophages (CD11b^+^CD80^+^CD86^+^), MDSCs (CD45^+^CD11b^+^GR-1^+^), and effector memory T (T_EM_) cells (CD8^+^CD44^+^CD62L^-^), and the cells were stained with corresponding antibody, including anti-FITC-CD3, anti-PE-CD4, anti-APC-CD8a, anti-FITC-CD11 b, anti-PE-CD80, anti-APC-CD86, anti-PE-CD45, anti-APC-CD 11b, anti-FITC-Gr-1, anti-FITC-CD8a, anti-PE-CD44, anti-APC-CD62L, and anti FITC-CD3, anti PE-CD4, anti APC-CD8a. The stained cells were analyzed via cytometry. Additionally, cytokine levels (IFNγ, TNFα, and IL6) were measured using the respective ELISA kits, following the manufacturer’s instructions. The detailed gate strategy was displayed in Figure S17-21.

**Statistical Analysis.** Statistical analysis was performed using GraphPad Prism 8.1. Data are presented as the mean values ± standard deviation (SD). One-way ANOVA with Tukey’s multiple comparisons test was employed for the statistical analysis of multiple groups. A significance threshold of *p <* 0.05 was considered statistically significant for all tests, ^⁎^*p <* 0.05, ^⁎⁎^*p <* 0.01, ^⁎⁎⁎^*p <* 0.001, ^⁎⁎⁎⁎^*p <* 0.0001, and ns denotes no significant difference.


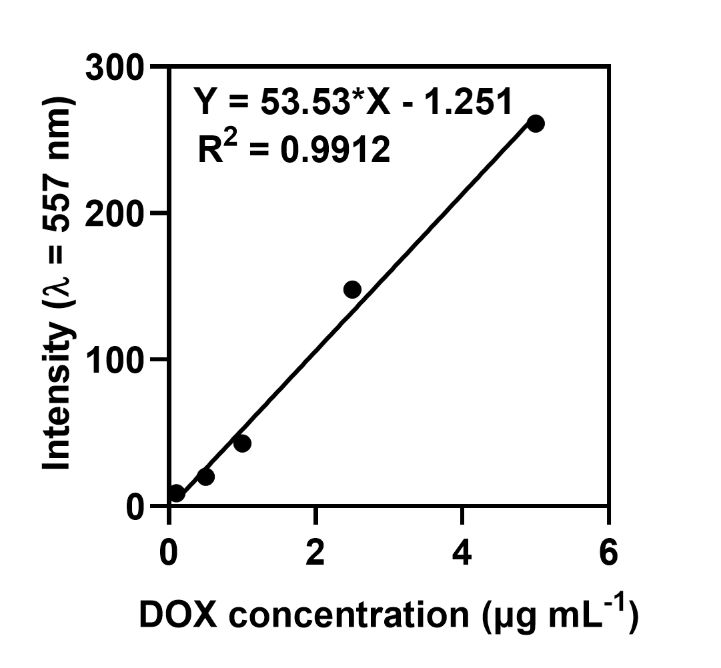


**Figure S1.** Fluorescence calibration curve of DOX.


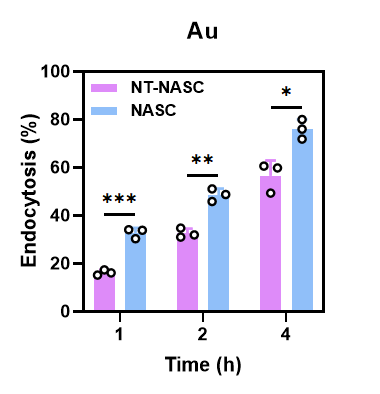


**Figure S2.** Evaluation of the Au content of 4T1 tumor cells with different treatments by ICP-MS detection. Data were performed as the mean ± SD (n = 8). ******p* < 0.1, *******p* < 0.01, ********p* < 0.001.


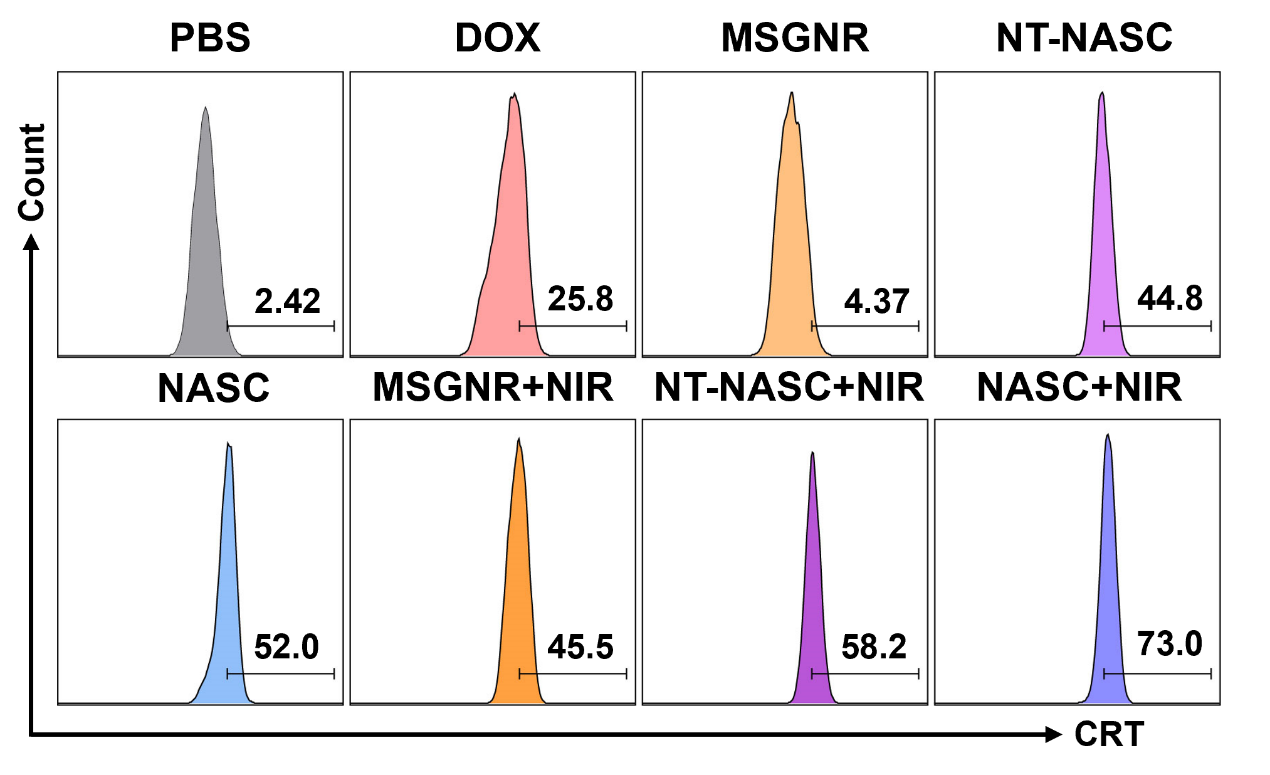


**Figure S3.** Flow cytometry analysis of CRT from 4T1 tumor cells treated with various NPs for 4 h.


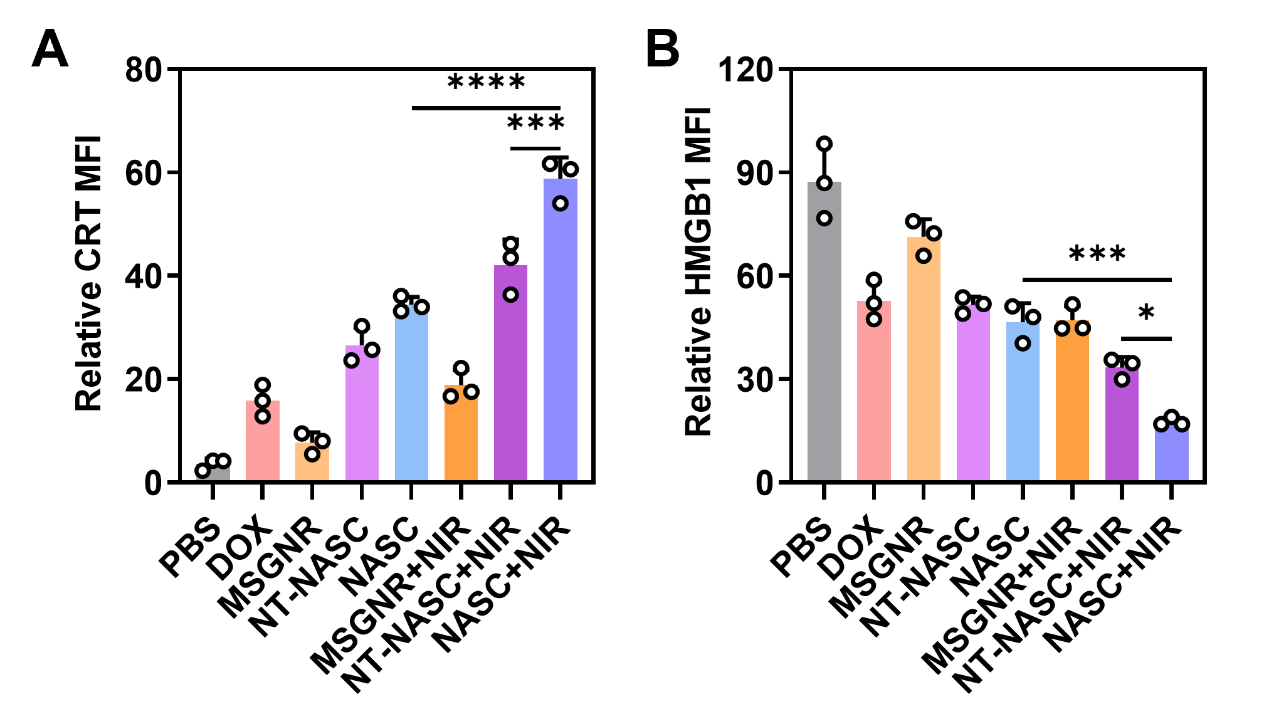


**Figure S4.** The corresponding quantification and comparison of fluorescence intensity A) CRT and B) HMGB1 using ImageJ software from four randomly selected images. Data were performed as the mean ± SD (n = 4). ******p* < 0.1, ********p* < 0.001, *********p* < 0.0001.


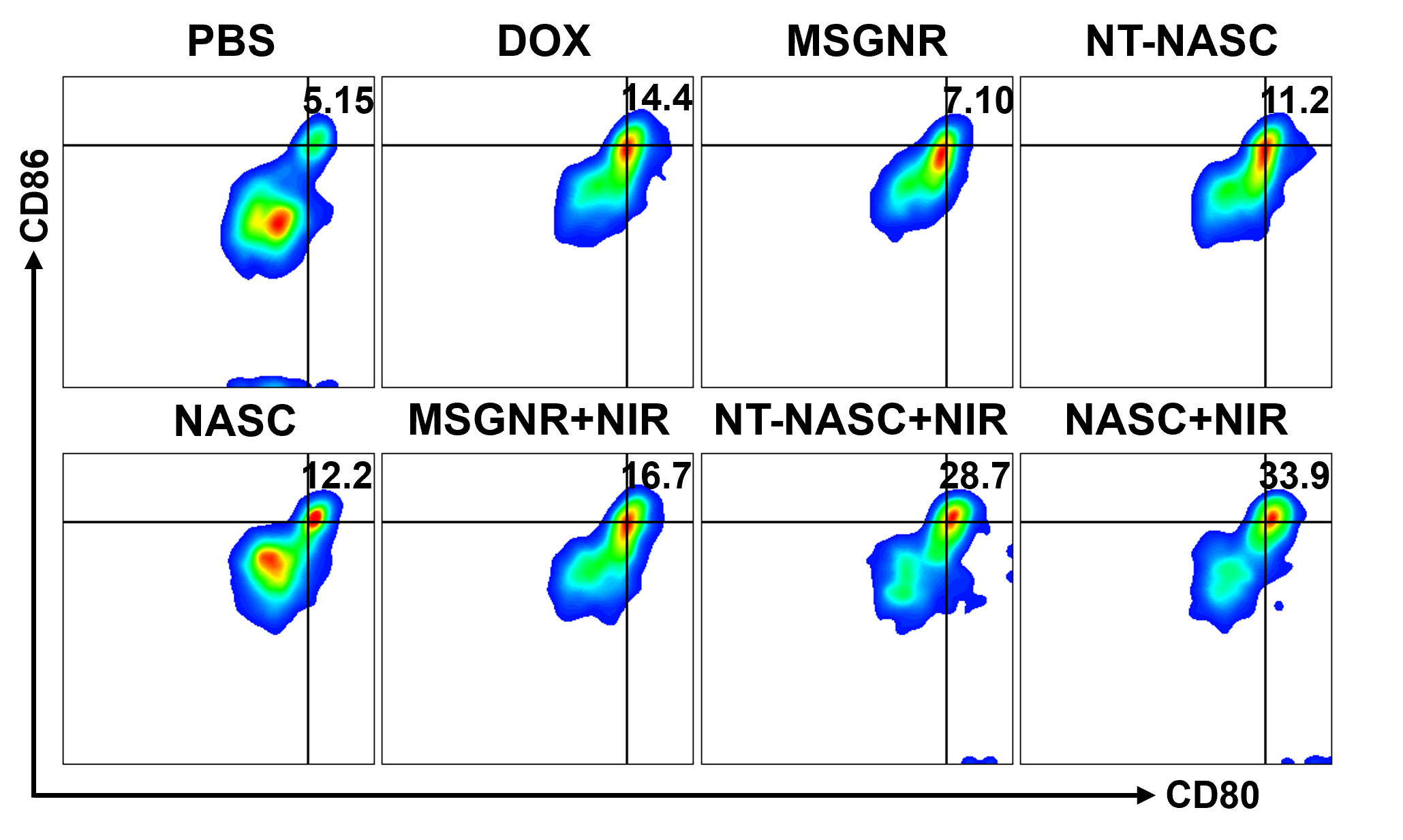


**Figure S5.** Representative flow cytometry plots of Matured DCs.


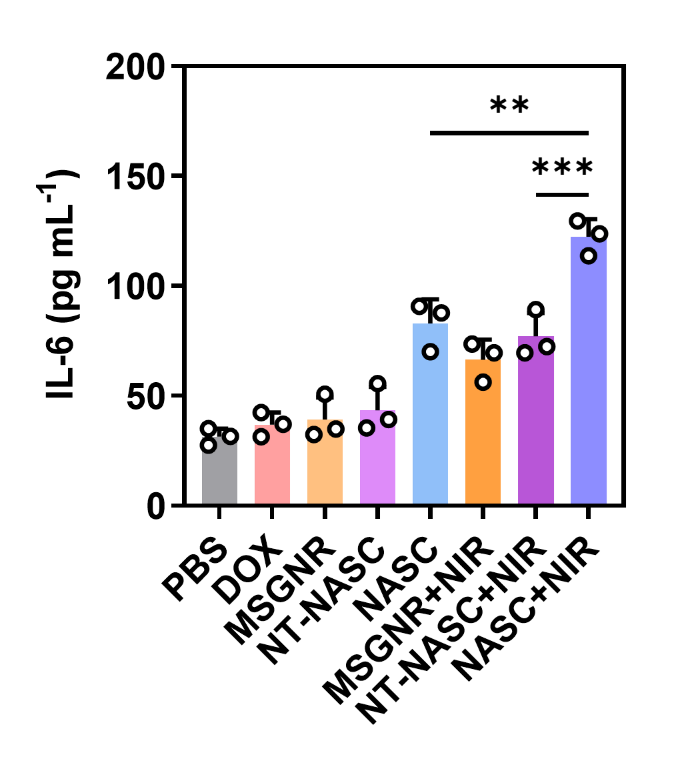


**Figure S6.** Blood content of IL-6 measured by ELISA assay. Data were performed as the mean ± SD (n = 3). *******p* < 0.01, ********p* < 0.001.


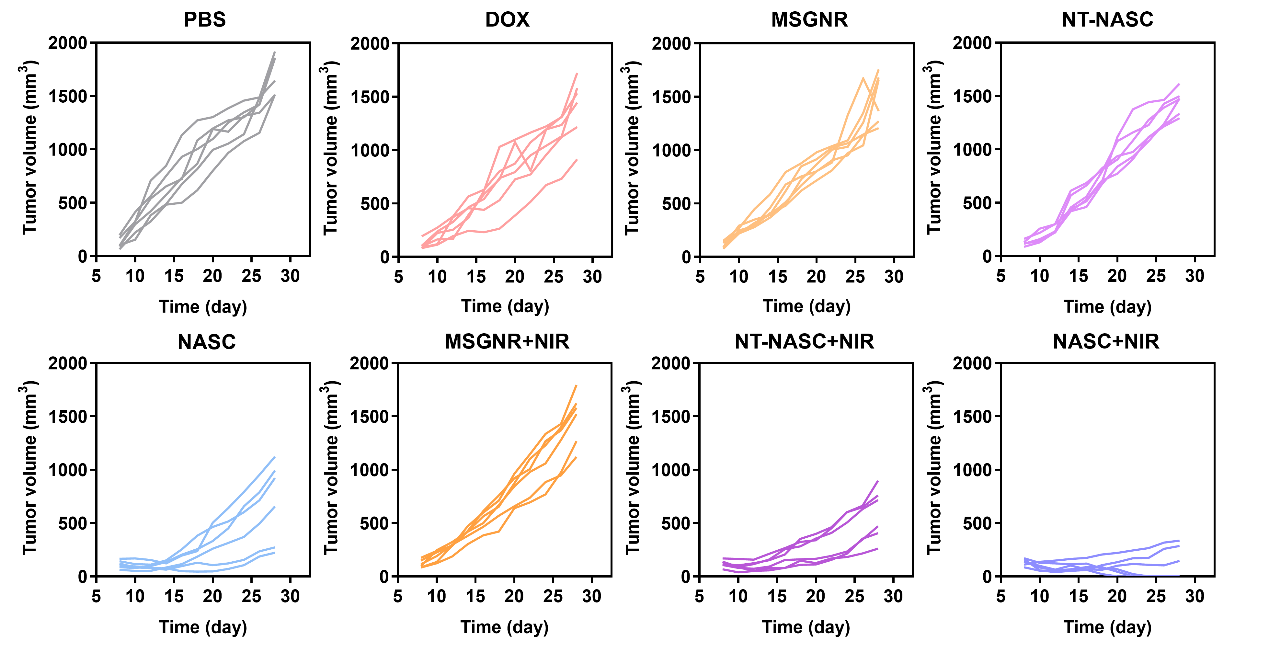


**Figure S7.** Individual tumor growth curves of unilateral tumor-bearing mice in different groups.


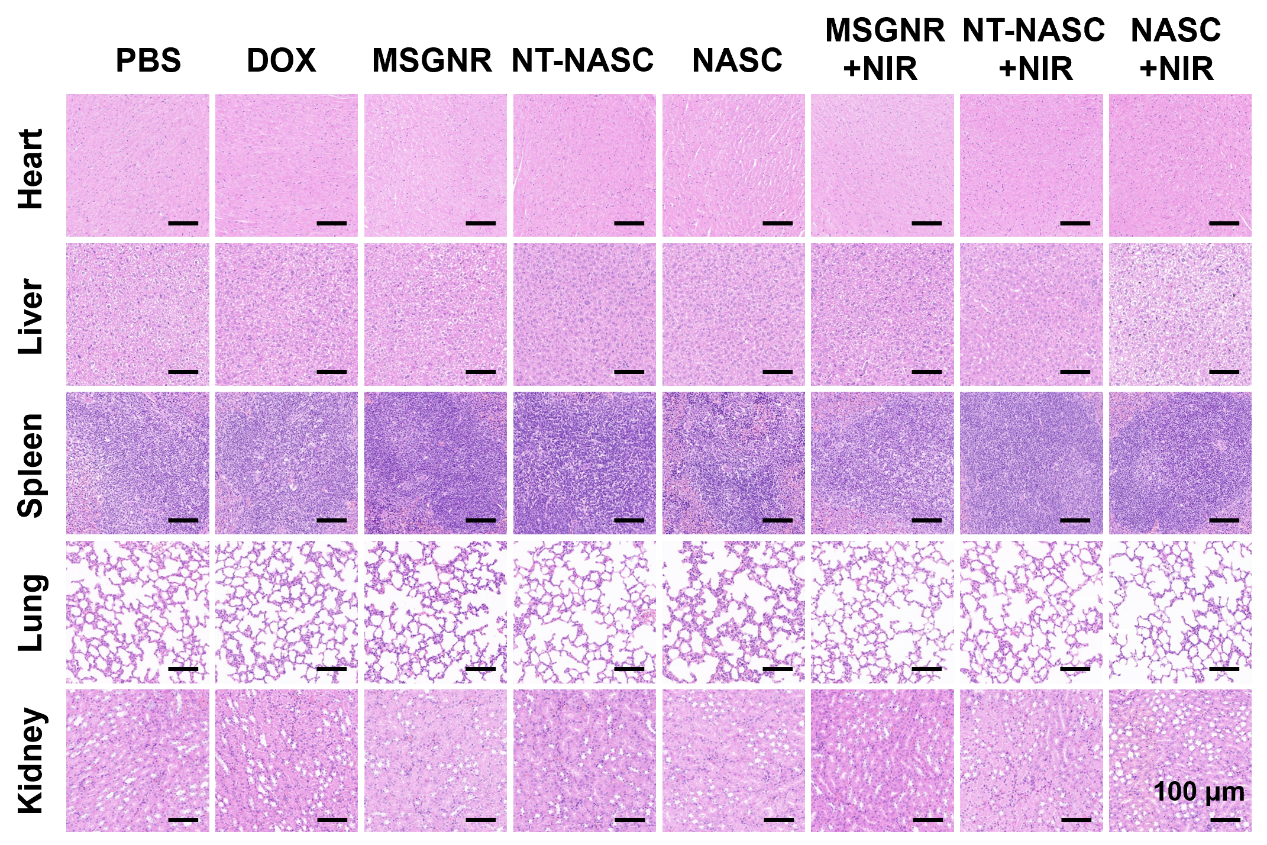


**Figure S8.** H&E staining of heart, liver, spleen, lung, and kidney after different treatments in the 4T1 tumor-bearing mice.


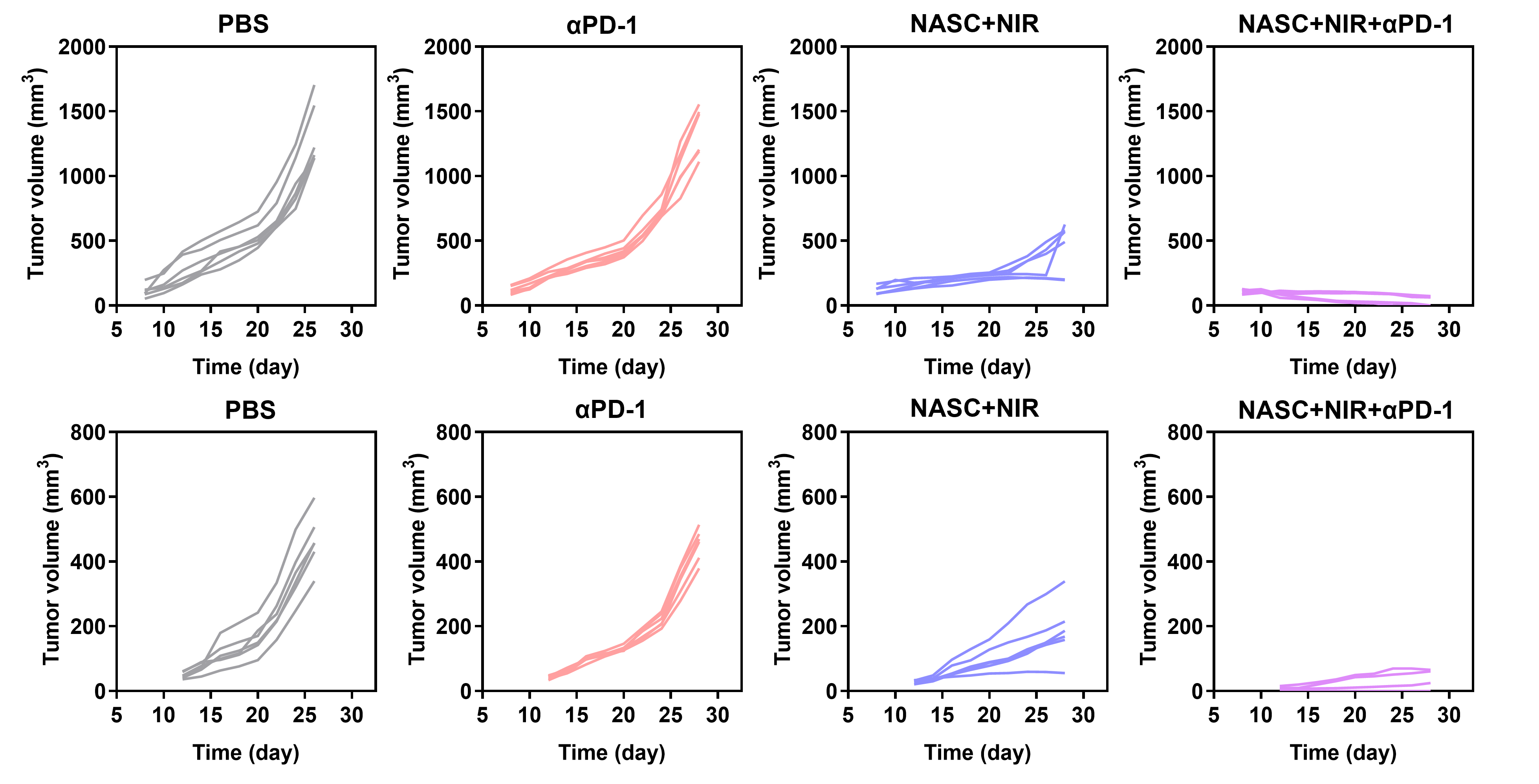


**Figure S9.** Individual tumor growth curves of bilateral tumor-bearing mice in different groups.


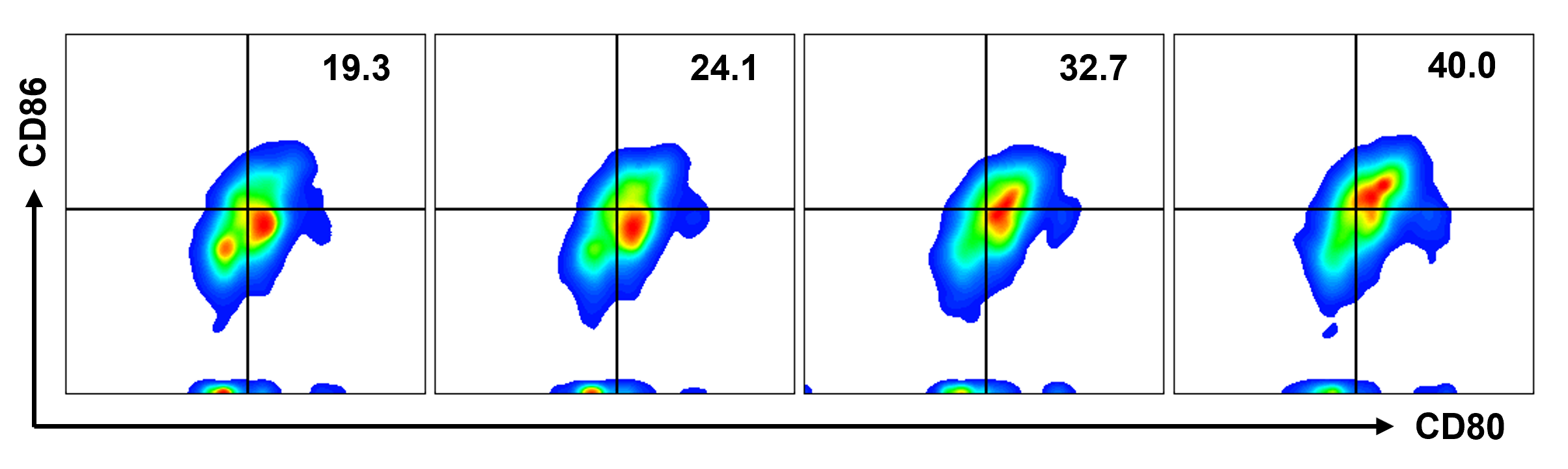


**Figure S10.** Representative flow cytometry plots of DCs maturation in lymph nodes in 4T1 tumor-bearing mouse models.


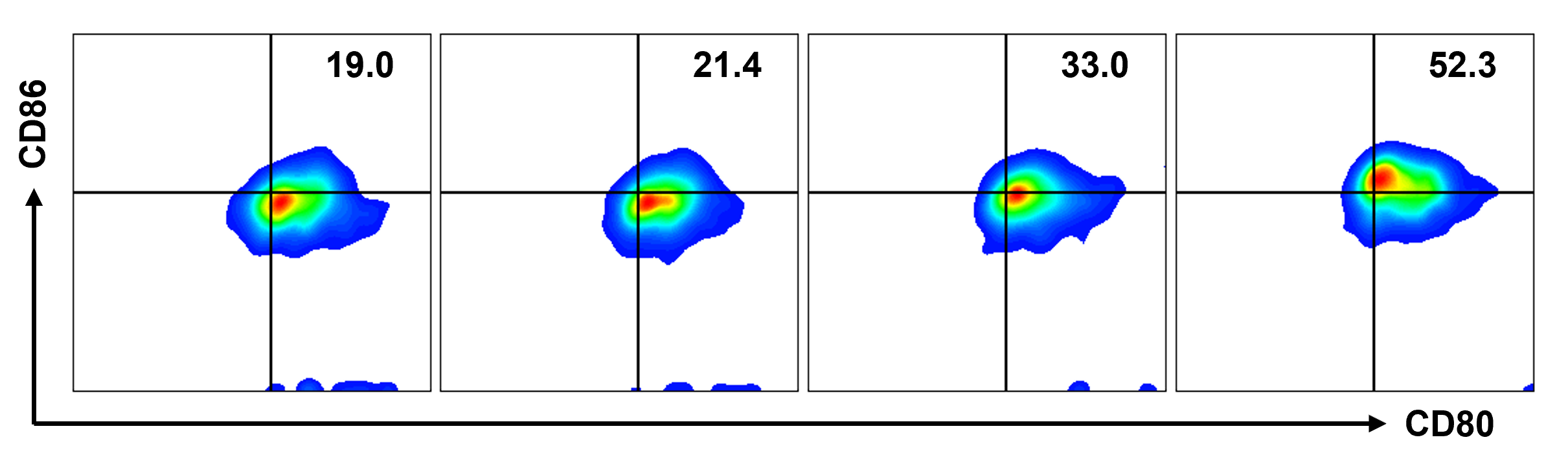


**Figure S11.** Representative flow cytometry plots of DCs maturation in primary tumors in 4T1 tumor-bearing mouse models.


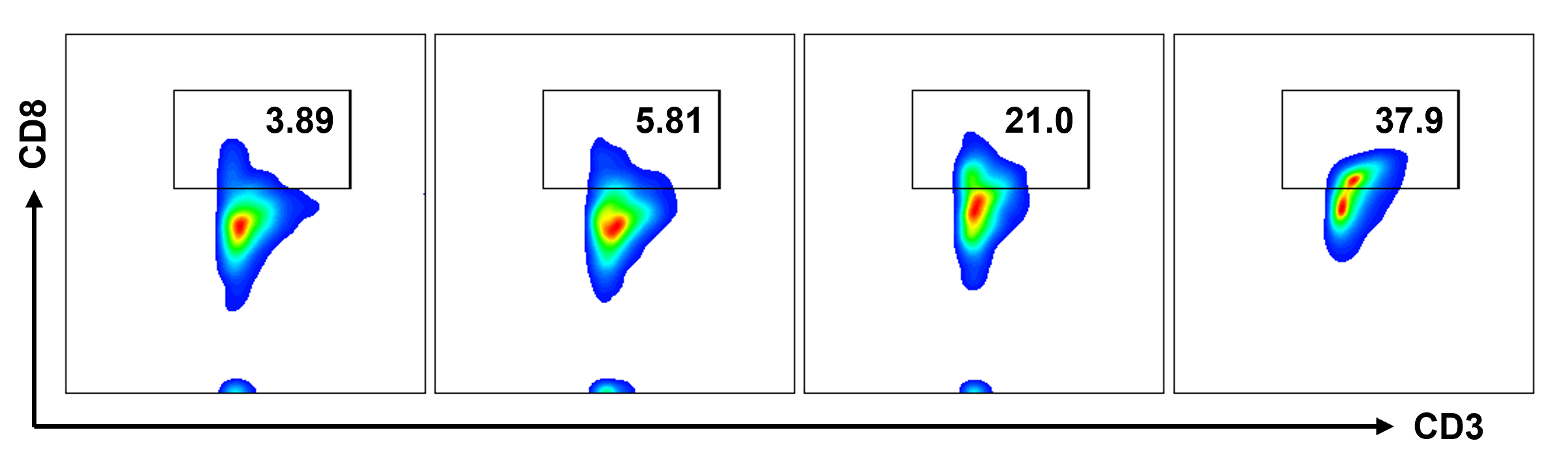


**Figure S12.** Representative flow cytometry plots of CD3^+^CD8^+^ T cells in primary tumors in 4T1 tumor-bearing mouse models.


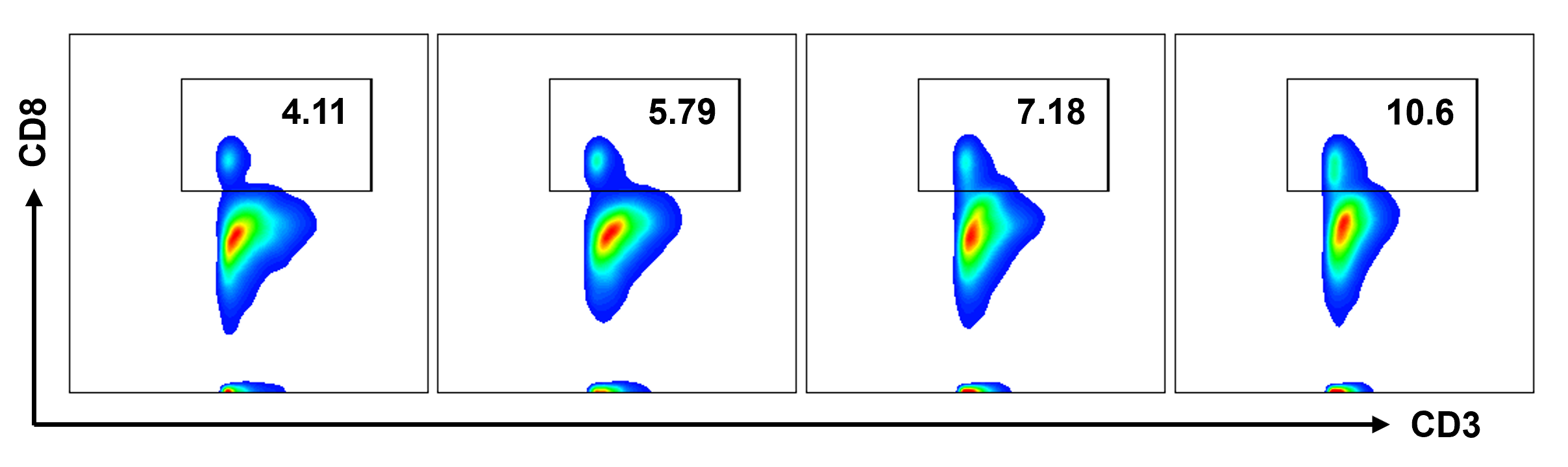


**Figure S13.** Representative flow cytometry plots of CD3^+^CD8^+^ T cells in distant tumors in 4T1 tumor-bearing mouse models.


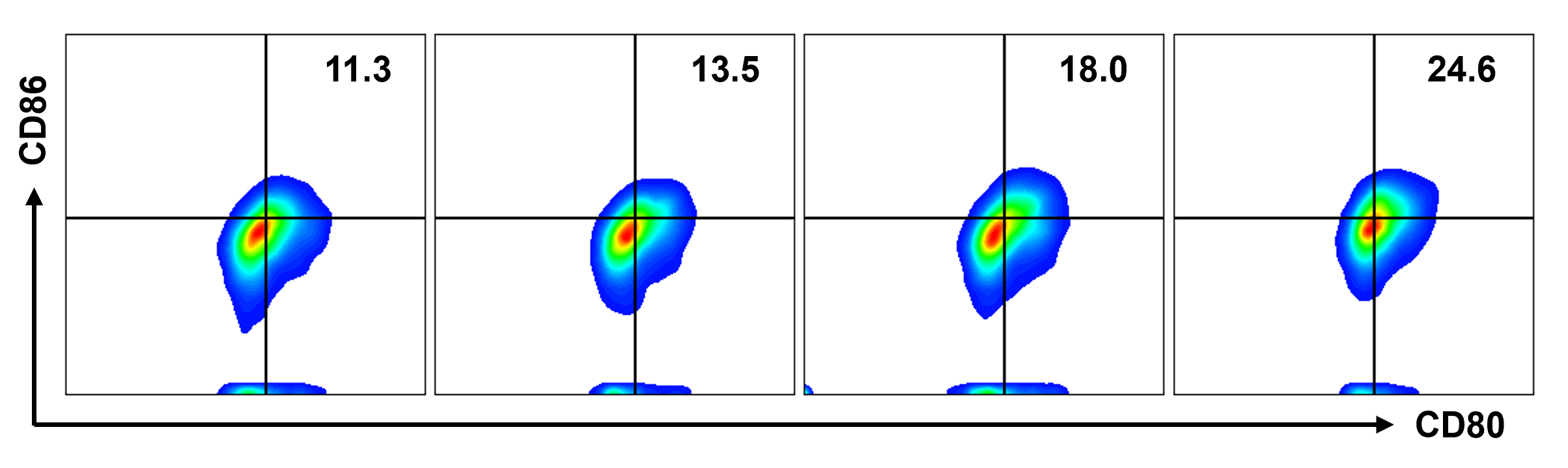


**Figure S14.** Representative flow cytometry plots of M1 macrophages in tumors in 4T1 tumor-bearing mouse models.


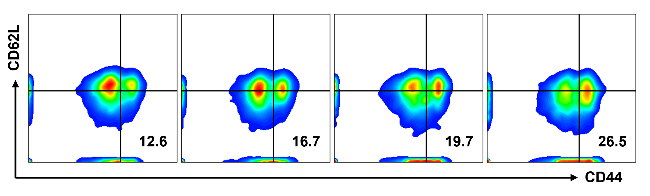


**Figure S15.** Representative flow cytometry plots of MDSC at primary tumor sites in 4T1 tumor-bearing mouse models.


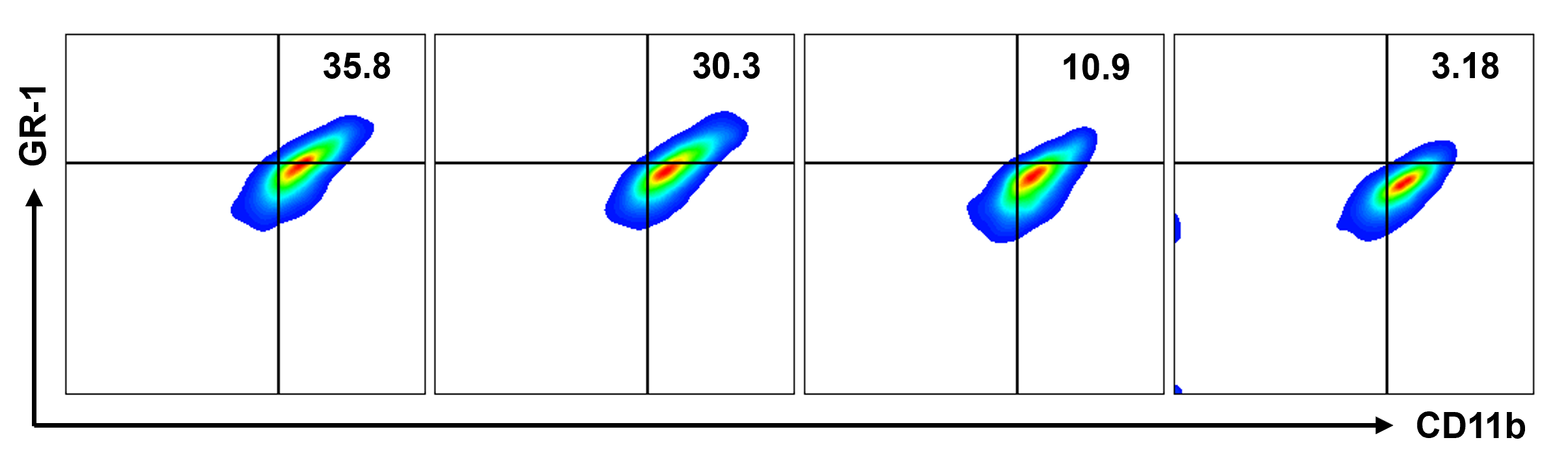


**Figure S16.** Representative flow cytometry plots of eﬀector memory T cells in spleens in 4T1 tumor-bearing mouse models.

**
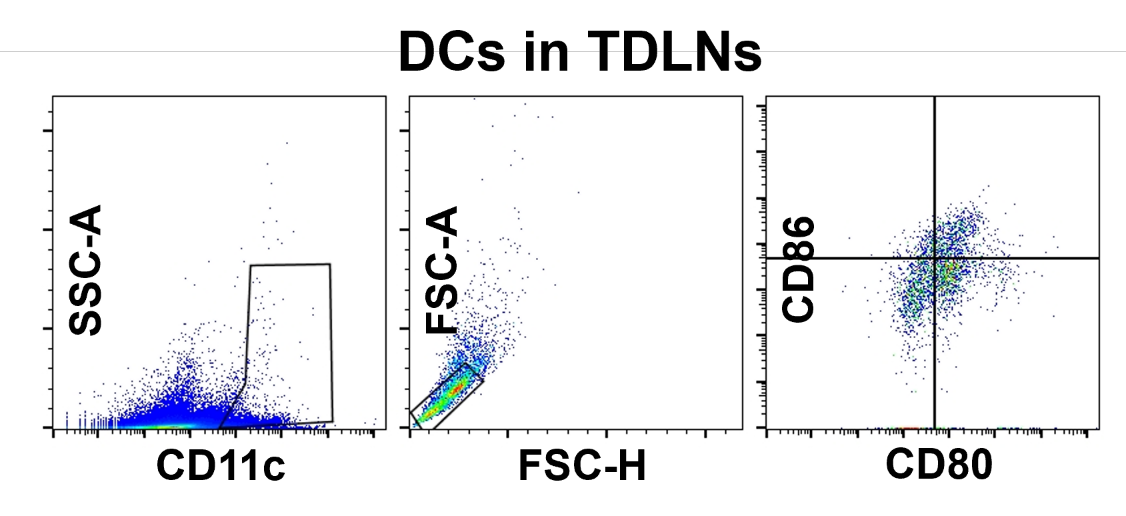
**

**Figure S17.** Gate strategy of DCs in TDLNs.


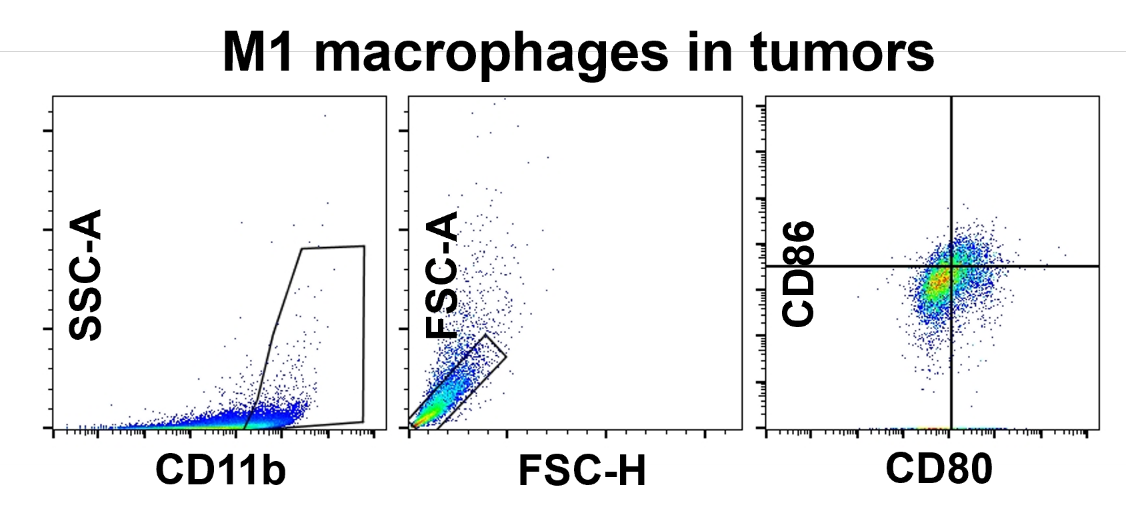


**Figure S18.** Gate strategy of M1 macrophages in tumors.


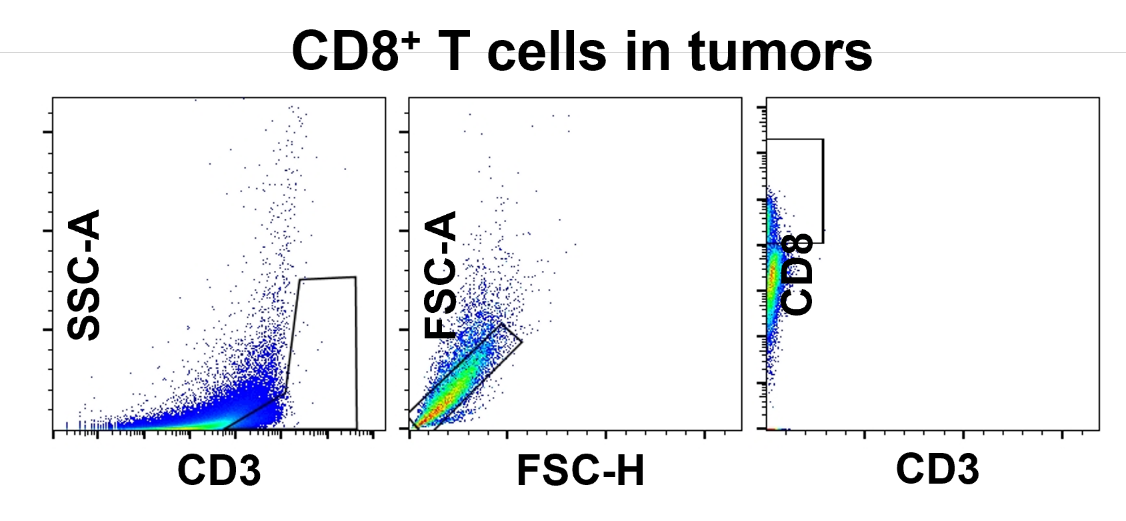


**Figure S19.** Gate Strategy of CD8^+^ T cells in tumors.


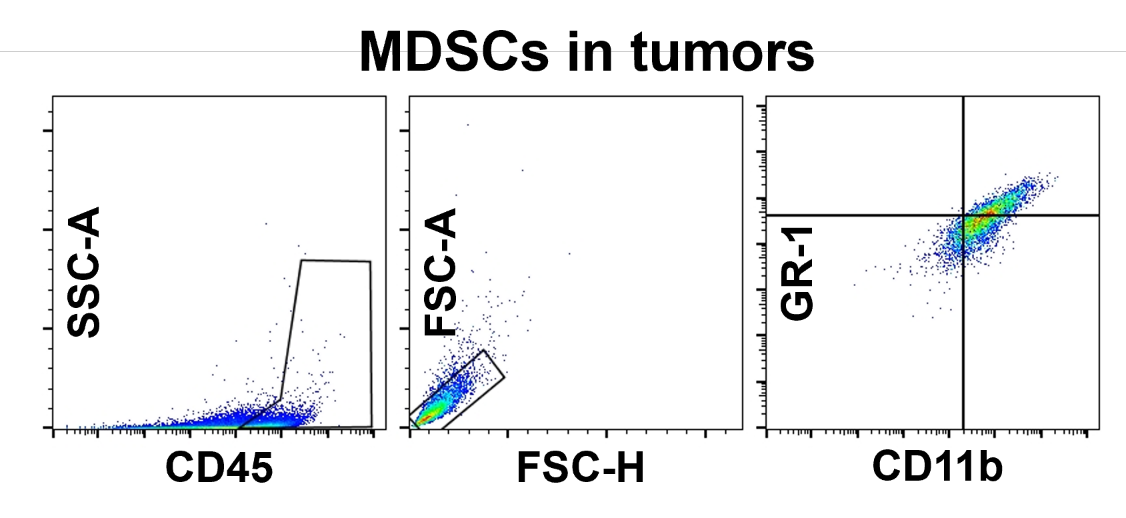


**Figure S20.** Gate Strategy of MDSCs in tumors.


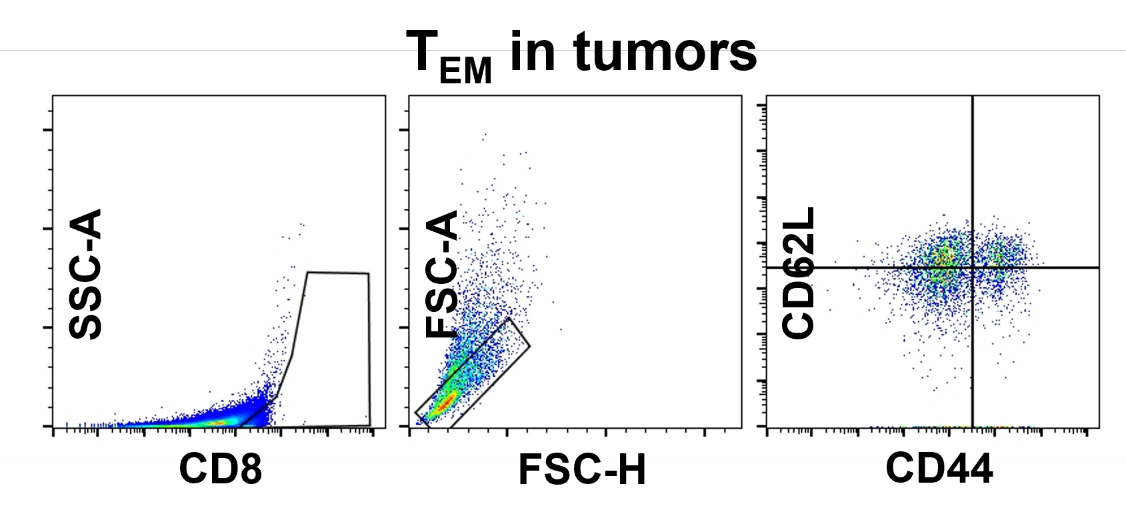


**Figure S21.** Gate Strategy of T_EM_ in tumors.
